# Beauty Is in the Eye of the Beholder: Proteins Can Recognize Binding Sites of Homologous Proteins in More than One Way

**DOI:** 10.1371/journal.pcbi.1000821

**Published:** 2010-06-17

**Authors:** Juliette Martin

**Affiliations:** Université de Lyon, Lyon, France; Université Lyon 1, IFR 128, CNRS, UMR 5086 Institut de Biologie et Chimie des Protéines (IBCP), Lyon, France; National Cancer Institute, United States of America and Tel Aviv University, Israel

## Abstract

Understanding the mechanisms of protein–protein interaction is a fundamental problem with many practical applications. The fact that different proteins can bind similar partners suggests that convergently evolved binding interfaces are reused in different complexes. A set of protein complexes composed of non-homologous domains interacting with homologous partners at equivalent binding sites was collected in 2006, offering an opportunity to investigate this point. We considered 433 pairs of protein–protein complexes from the ABAC database (AB and AC binary protein complexes sharing a homologous partner A) and analyzed the extent of physico-chemical similarity at the atomic and residue level at the protein–protein interface. Homologous partners of the complexes were superimposed using Multiprot, and similar atoms at the interface were quantified using a five class grouping scheme and a distance cut-off. We found that the number of interfacial atoms with similar properties is systematically lower in the non-homologous proteins than in the homologous ones. We assessed the significance of the similarity by bootstrapping the atomic properties at the interfaces. We found that the similarity of binding sites is very significant between homologous proteins, as expected, but generally insignificant between the non-homologous proteins that bind to homologous partners. Furthermore, evolutionarily conserved residues are not colocalized within the binding sites of non-homologous proteins. We could only identify a limited number of cases of structural mimicry at the interface, suggesting that this property is less generic than previously thought. Our results support the hypothesis that different proteins can interact with similar partners using alternate strategies, but do not support convergent evolution.

## Author Summary

Interaction between proteins is a fundamental process, generic to most biological pathways. The increasing number of protein–protein complexes with atomic data should help us to understand the major factors that guide protein interactions. In particular, a number of examples are available of similar proteins that interact with proteins that are very different in terms of structure and function. An intuitive hypothesis to explain the ability of these different proteins to recognize the same partner is that they display the same local region for interaction, in other words, they imitate the same binding site. Here, we quantify the similarity between these putatively mimicking binding sites. We show that it is not statistically significant. We confirm this observation on the small sets of evolutionarily conserved residues. Our results suggest that different proteins that bind the same protein do not imitate binding sites, but probably target specific locations or residues at the binding site.

## Introduction

Protein-protein interaction is the basis of numerous biological functions, such as immune response, supra-molecular assembly, enzymatic reactions, and many more. Understanding the way proteins interact is thus a fundamental challenge. The collection of all protein-protein interactions, the interactome, is also of great importance for drug discovery [1]. Given their variety and often transient nature, the number of protein-protein complexes for which crystallographic structures are available is very limited compared to the number of individual protein structures in the Protein Data Bank [2]. But even with this limited amount of data, the observation of available complexes has helped to decipher some rules for protein-protein interactions. Among the properties playing a role in this process, hydrophobicity was suggested as a major factor by Chothia and Janin in their pioneering work [3]. Other characteristics that are important for interaction, or that can be used to describe binding sites, include size, shape complementarity, residue propensity and packing density [4]–[6]. Sequence conservation is also widely acknowledged as an important feature of protein-protein recognition [7], [8]. Additional studies have further refined the picture. For example, binding sites are organized as a core of buried residues, surrounded by a rim of accessible residues, with distinct amino-acid composition and evolutionary conservation patterns [9], [10]. Nicola and Vakser found that the binding site is, on average, closer to the center of mass of the protein compared to other surface residues [11]. Different types of complexes (e.g. homo- or hetero-dimers, transient or permanent) display different properties [8], [12], [13].

A notable element to understand the mechanism of protein-protein interaction is the existence of hot spots, residues that make major contributions to the binding energy, see for example [14] for a review. In their landmark paper, Bogan and Thorn showed that hot spots are localized at the center of interfaces, and surrounded by a ring of energetically unimportant residues, that protect them from the solvent [15]. This is called the O-ring theory, and has been recently refined by Li and Liu [16].

Several groups have addressed the question of the evolutionary conservation of protein-protein binding sites and binding modes. At first found to be insignificant [17], the conservation of interface residues has since been shown to be more pronounced in biological interfaces than in crystallographic ones or over the rest of the protein surface [18], [19]. This change of viewpoint probably comes from the increase of available data, as well as the variety of computational approaches developed to quantify conservation, and also the fact that some proteins have multiple interfaces [20]. The link between evolutionary conservation and hot spots is unclear: overall difference in conservation between hot spot and non hot spot residues is marginal [21], [22]; conservation used in combination with other features has been found to improve hot spot prediction in [21] but not in [22]. From a more macroscopic point of view, complexes that share more than 35% identity commonly share similar structures and interaction modes [23]. The localization of a binding site on a protein is preserved within SCOP families, but not necessarily at the super-family level [24], [25].

Another important notion we want to introduce here is the existence of promiscuous proteins. Promiscuity, also called multi-functionality or moonlighting, denotes the ability of one protein to perform distinct functions, see reviews [26], [27]. A recent review reveals that promiscuity is not as rare as previously thought [28]. Examples notably include transcription regulatory proteins that can act as transcription coactivators or enzymes [29]. More generally, a promiscuous protein can interact with different partners. These multi-partner proteins have been the subject of dedicated studies. For example, Keskin et al have shown that multi-partner protein interfaces have original properties: they are smaller and less packed than other interfaces [30]. A recent survey of proteins with multi-binding protein interfaces involving 97 pairs of complexes from 49 protein families revealed that multi-binding interfaces are not more conserved than other interface sites [31]. The energetic determinants of multi-partner proteins have also been addressed: interactions involving specific binding sites display higher affinities than those of promiscuous binding sites [32]. In an earlier work, Humphris and Kortemme employed a computational design procedure to optimize the binding site of 20 multi-specific proteins, so that they maintained interactions with all their known partners (multi-constraint protocol) or with each partner separately (single-constraint protocol) [33]. For half of the tested cases, they obtained different results using the single and the multi-constraint protocol, suggesting that promiscuous binding sites are optimized for multi-specificity in such a way that each partner prefers its own set of residues on the binding site. A recent analysis using state-of-the-art computational methods applied on calmodulin, whose structure is available in complex with 16 different targets, confirmed this hypothesis [34]. These analyzes focused on the common, promiscuous binding sites, but not on the binding sites of the multiple partners.

The fact that a promiscuous protein can bind to different partners using the same binding site is puzzling, but also of outstanding interest to further understand the mechanisms of protein-protein interactions. Does this observation imply that radically different proteins possess similar binding sites in order to recognize a single promiscuous protein? At first sight, it might seem hopeless to look for similar binding sites on non-homologous proteins that differ in structure, function and ancestry. However, the literature is rich in examples of approaches employing - or searching for - such local similarities between unrelated proteins. This is the case for at least three distinct targets: catalytic sites, ligand binding sites and protein-protein binding sites. In the case of catalytic sites, the well-known example of the catalytic triad pattern, found in diverse serine proteases, has motivated a number of developments [35]–[40]. Concerning ligand binding sites, their generic nature among unrelated proteins has lead to the development of many comparison approaches [41]–[50]. Lastly, for protein-protein interactions, the similarity between proteins with very different folds has been investigated in several studies. An important corpus of work on this problem comes from Nussinov and colleagues. Using geometric hashing, they created clusters of similar interfaces based on the C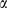 geometry [51] and found clusters with similar interfaces despite different overall structures, as well as clusters where only one side of the interface was conserved [52], [53]. Shulman-Peleg et al. subsequently developed the I2I-SiteEngine software, dedicated to structural alignment of protein-protein interfaces, based on the similarity of their physico-chemical properties and shapes [54], [55]. These observations have been applied to the prediction of protein-protein interactions, with the development of the PRISM database [56], [57], and to structural alignment of protein-protein interfaces, with the MAPPIS web server [49]. Other groups have also investigated this question. Zhu et al. proposed the Galinter method, based on the representation of interfaces by vectors representing van der Waals interactions and hydrogen bonds between protein chains, allowing binding site comparison using graph algorithms [58]. Very recently, Konc et al. have proposed ProBis, a graph-based method for binding site prediction [59]. Convergent evolution thus seems to exist also for protein-protein interactions [60], [61].

In this paper, we analyze a set of protein-protein complexes involving homologous proteins in interaction with different partners. These examples come from an analysis of PDB complexes in terms of SCOP domains, and are stored in the ABAC database [61]. Truly speaking, these complexes do not illustrate promiscuity, since they involve homologous (same SCOP family) rather than identical proteins. We therefore term this promiscuous binding at the family level. Our goal is to understand how unrelated proteins can bind to similar targets. In particular, we looked for similar atoms or groups of atoms at the interface of different proteins that bind similar partners and assessed the significance of the similarity between interfaces using a bootstrap procedure. We also considered evolutionarily conserved residues, as they probably play a dominant role in the binding. Our results support the hypothesis that different partners often interact with a single partner using alternate strategies, and do not point to convergent evolution.

## Results

**Figure 1:**
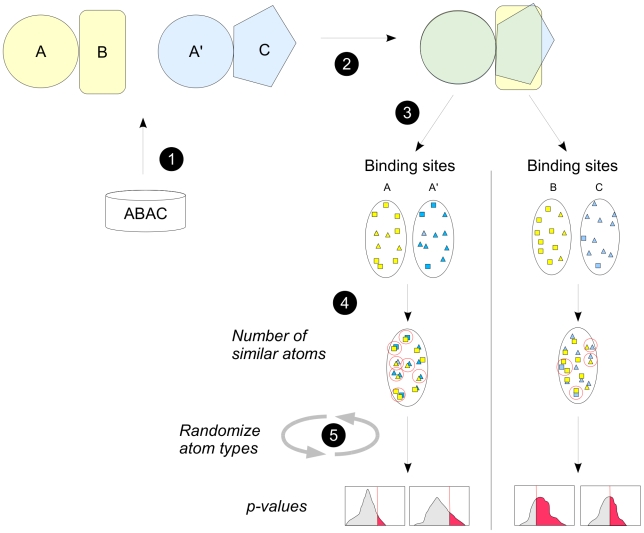
Schematic representation of the methodology. (1) pairs of complexes in which homologous proteins A and A′ are seen in interaction with two unrelated proteins B and C are retrieved from the ABAC database; (2) homologous proteins A and A′ are superimposed using Multiprot; (3) the analysis is restricted to protein-protein interaction binding sites, and carried out separately for A/A′ and B/C sides; (4) the number of similar atoms is computed after superimposition of the binding sites: here, two different types of atoms are represented by squares and triangles; (5) random interfaces are created by randomizing the atom types, in order to obtain random distributions and to compute p-values.

The overall methodology used to assess the similarity at protein-protein interfaces is summarized in Figure 1 detailed in the Materials and Methods section.

**Figure 2:**
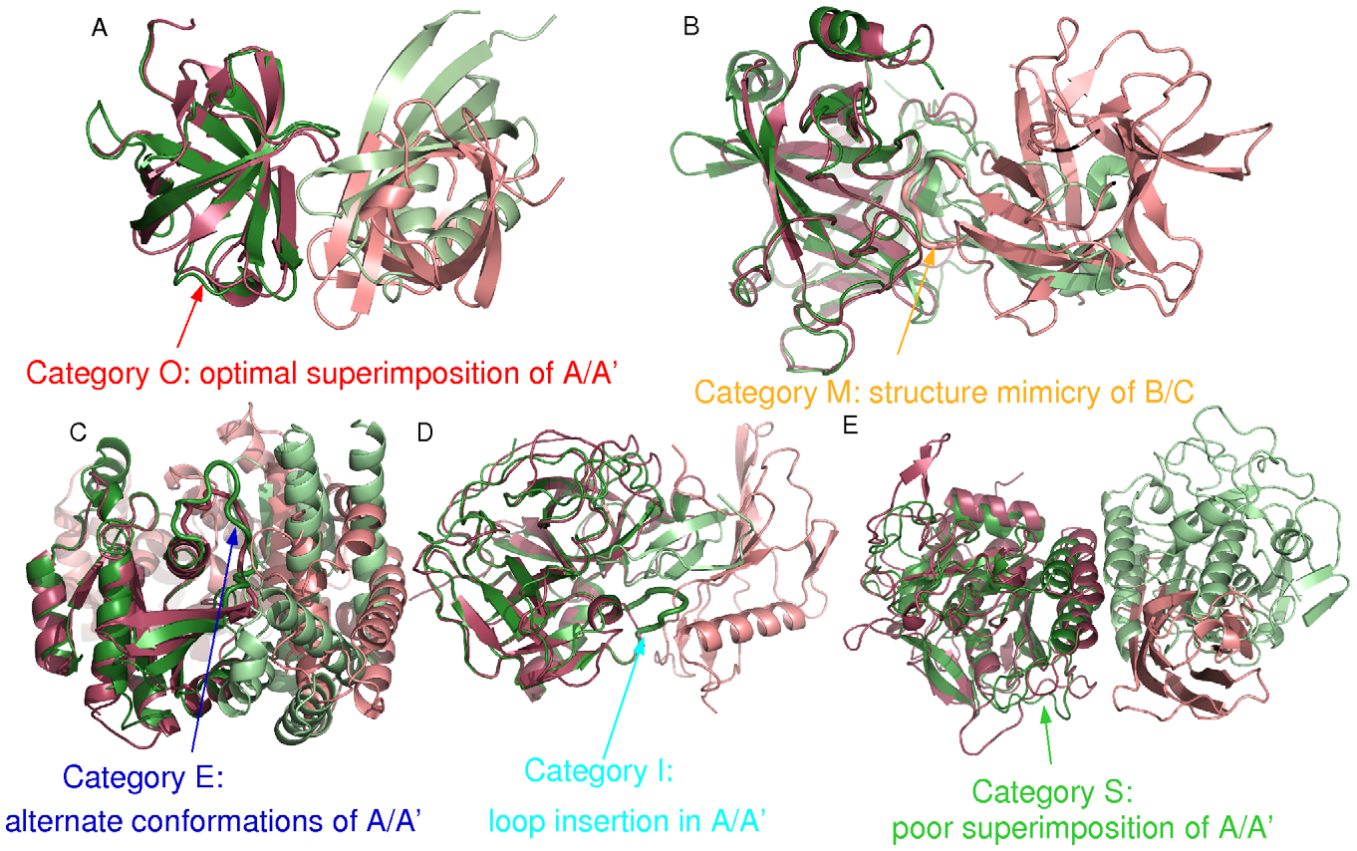
The five categories of ABAC pairs. For each pair of complexes, one structure is displayed in pink and the other in green, with the superimposed A/A′ domains on the left side and the B/C domains on the right side. Images are generated using Pymol [78]. Structural mimicry, alternate loop conformations and residue insertion/deletion are highlighted by thicker representations. Hereafter, complexes are named by their PDB code (first four letters), combined with the identifiers of interacting chains (last two letters). A: category O, PDB structure 1dg1_HG (dimer of domain 2 of elongation factor Tu of *E. coli*) *versus* PDB structure 1g7c_AB (domain 2 of elongation factor eEF-1 alpha from *S. cerevisiae* complexed with guanine nucleotide exchange factor domain from elongation factor-1 beta), B: category M, PDB structure 1avw_AB (trypsin from pig complexed with soybean trypsin inhibitor) *versus* PDB structure 1fak_BD (human coagulation factor VIIa complexed with bovine pancreatic trypsin inhibitor), C: category E, PDB structure 1wq1_RG (human cH-p21 Ras protein complexed with p120GAP domain) *versus* PDB structure 1gzs_AB (human CDC42 complexed with GEF domain of SopE toxin from *S. typhimurium*), D: category I, PDB structure 1bui_AC (catalytic domain of human plasmin complexed with staphylokinase from *S. aureus*) *versus* PDB structure 1gl0_BA (bovine chymotrypsinogen complexed with protease inhibitor PMP-D2V from *L. migratoria*), E: category S, PDB structure 1p8j_HE (N-terminal domain of murine furin complexed with C-terminal domain of furin) *versus* PDB structure 1ic6_AB (dimer proteinase K from *T. album*).

**Figure 3:**
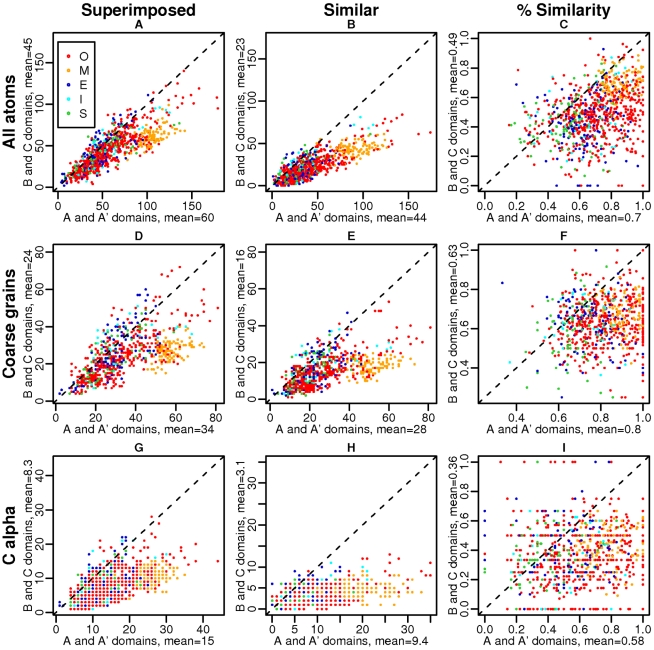
Similarity at protein-protein interfaces in ABAC pairs. First row: all-atom representations, second row: coarse-grain representations, third row: C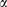 representations. First column: number of superimposed elements on A/A′ *versus* B/C side, second column: number of similar elements on A/A′ *versus* B/C side, third column: fraction of similar elements on A/A′ *versus* B/C side.

The ABAC pairs are classified into five categories on the basis of the quality of the superimposition between the two complexes, as illustrated in Figure 2. The first two categories, O and M (see Figures 2A and B) represent ideal cases to study promiscuous binding at the family level, with A/A′ domains having very similar structures which are easily superimposed. These two categories, encompassing 299 ABAC pairs, will be privileged in analyzing the similarity of binding sites, since the interfaces of A/A′ domains are well superimposed and the subsequent analysis of B/C binding sites is thus expected to be less noisy. Furthermore, the M category has the interesting particularity of exemplifying interface mimicry: domains B and C, although they have different global folds, display strikingly similar structures at the interface. It should be noted that among the 53 ABAC pairs in the M category, only 3 different SCOP families of A/A′ domains are represented, see Table 1. Eukaryotic proteases (family 50514) are seen in 49 pairs, subtilisin-likes (family 52744) in three pairs, and interleukin 8-like chemokines (family 54118) in one pair. Pairs of the category M are thus largely dominated by eukaryotic proteases complexed with various inhibitors, which, as shown in Figure 2 B, display a protruding/interwound geometry, with the B/C mimicry interfaces embedded in the A/A′ domain. This introduces a significant bias in interface size, with more residues involved in the interface on the A/A′ side than on the B/C side, see Figure 3 and Table 3 in Text S1. The three other categories, E, I and S (see Figures 2 C, D and E), illustrate three degrees of difficulty in A/A′ superimposition, with, respectively, alternate conformations in the binding site, residue insertion/deletion in the binding site, and overall poor structural similarity, which might alter the analysis of interface similarity.

**Table 1:** SCOP family diversity in the data set.

Category^1^	Nb^2^	Fam(A/A′)^3^	Fam(B/C)^4^
O	246	77	188
M	53	3	18
E	63	16	57
I	21	13	34
S	50	21	67
Total	433	105	241

1: Category of the pairs of complexes.

2: number of pairs.

3: number of distinct SCOP families for A/A′ domains.

4: number of distinct SCOP families for B/C domains.

In the rest of the paper, we present a quantitative analysis of similarity at protein-protein interfaces in ABAC pairs, and then evaluate its significance against a random model. We also survey the similarity of interfaces in terms of evolutionarily conserved residues.

### Quantification of similarity

We first compute the number of similar elements - atoms, pseudo-atoms or residues - in each partner of the protein complexes after structural superimposition of the common partners A and A′. Domains A and A′ are from homologous domains from the same SCOP family. Consequently, we expect a good level of similarity between them. However, since such similarity results from divergence from a common ancestor and fold conservation, it does not necessarily imply that the similar elements are key determinants for the protein-protein interaction. Domains B and C are from different SCOP superfamilies. They thus have very different structures, but a common ability to bind to the same, or, at least, a similar partner. Similar elements between B and C could thus be a sign of evolutionary convergence to a given binding motif, or indicate which functional groups are essential for the binding.

**Figure 4:**
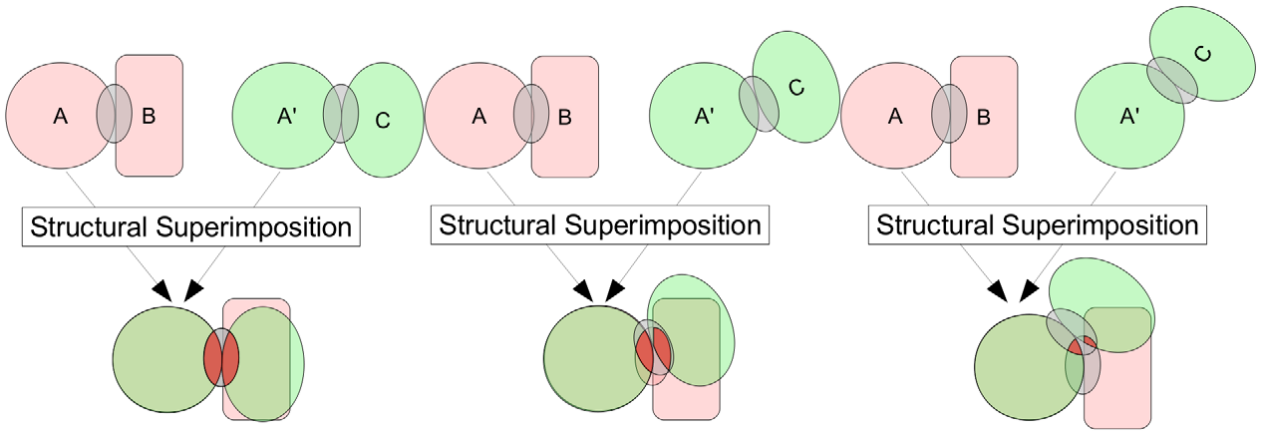
Schematic illustration of ABAC pairs. Domains A and A′, from the same SCOP family, interact with B and C from different SCOP superfamilies. The overlaps of binding sites, indicated by gray ellipses, are highlighted in red. The three figures illustrate three levels of spatial overlapping between binding sites. By construction, the size of the overlap on the A/A′ side is greater than on the B/C side.

**Figure 5:**
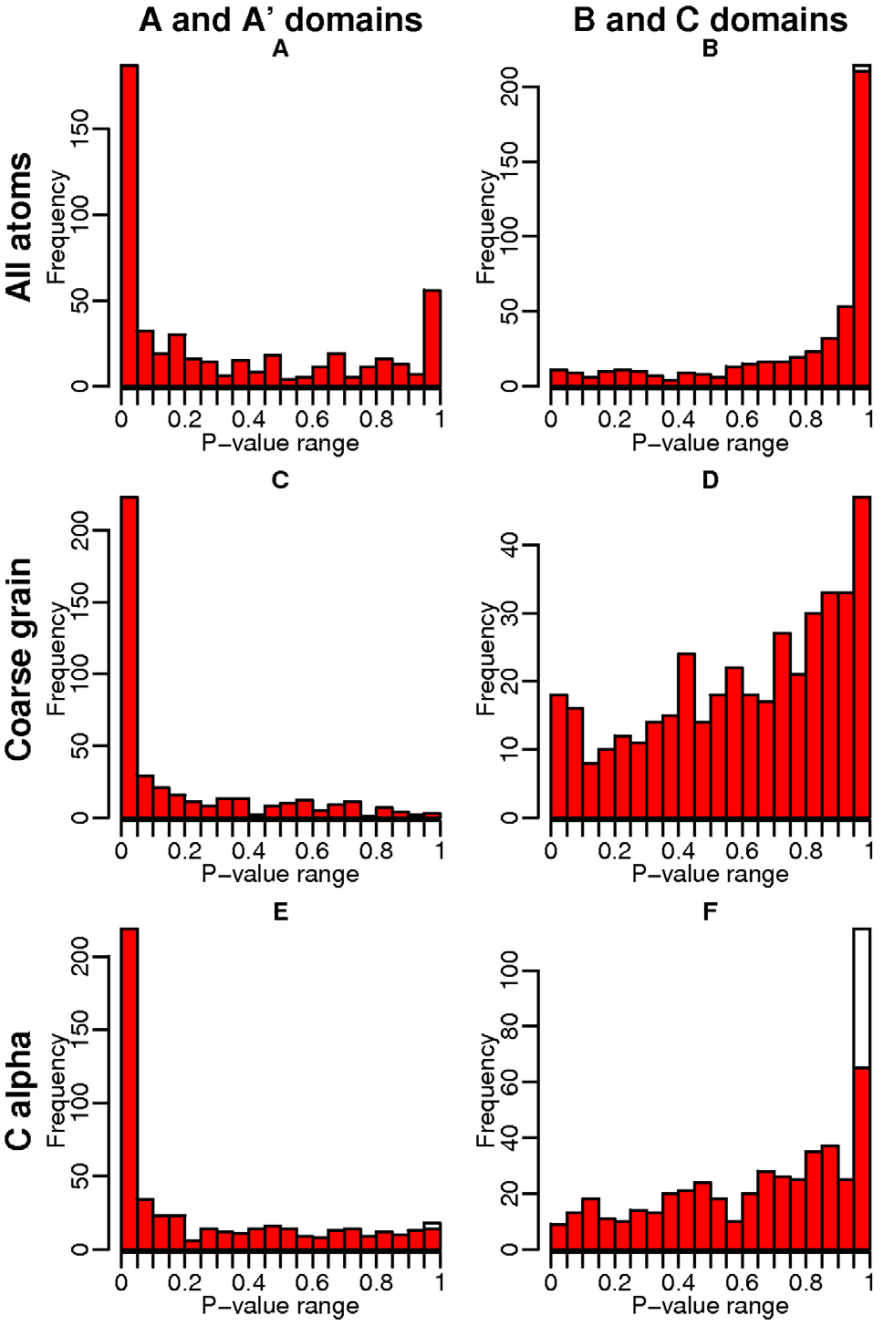
Distribution of similarity P-value at protein-protein interfaces of ABAC pairs of the category O. First row: all-atom representations, second row: coarse-grain representations, third row: C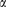 representations, first column: P-values of the A/A′ domains, second column: P-values of the B/C domains. White bars correspond to a number of similar elements equal to zero, which, by definition, yields a P-value equal to 1, since the random model cannot give a number of similar residues lower than zero.

**Figure 6:**
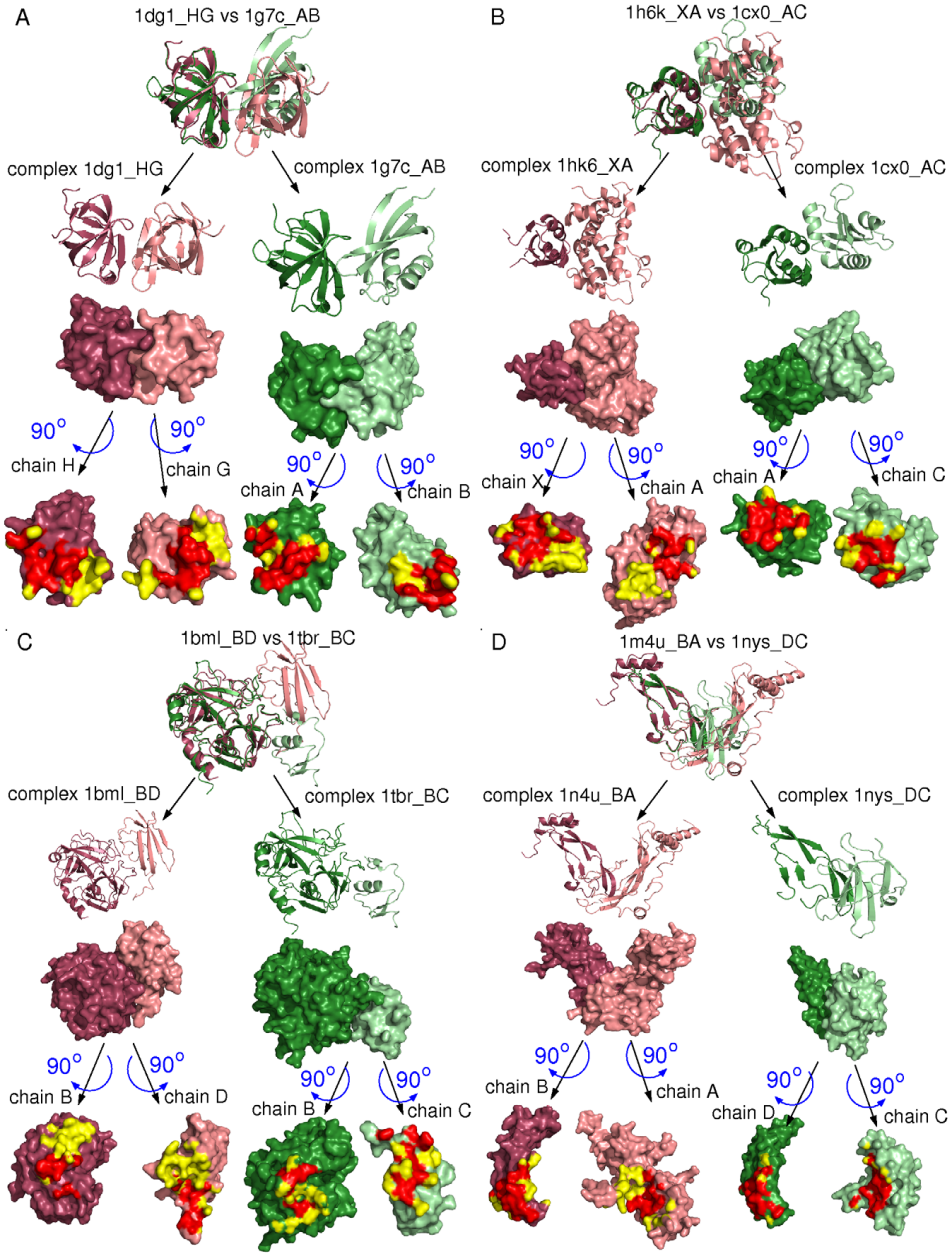
Extent of overlap of interfaces in ABAC pairs. Overlap of binding sites is highlighted in red. Residues involved in one of the binding sites but out of the overlap are highlighted in yellow. The four pairs of complexes belong to the O category.

**Figure 7:**
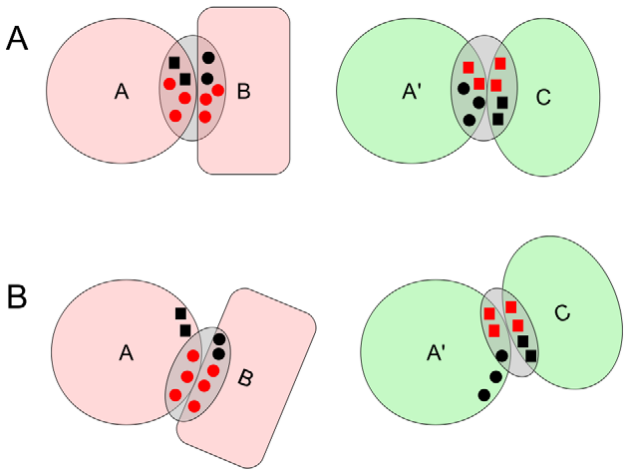
Schematic view of promiscuous protein-protein binding at the family level. Atoms/residues at the interfaces are symbolized by small squares and circles. The preferred atoms/residues in each complexes are highlighted in red, they are the key determinant of the complexes. A: different binding partners B and C interact at the same binding site of the similar proteins A and A′, but use their own set of atoms/residues. B: different binding partners B and C use atoms/residues out of the common binding site of A/A′. In both cases, binding sites of A and A′ are similar, but the alternate binding strategies can result in no similarity between B and C binding sites.

Figure 3 presents the number of superimposed and similar elements at the interface in the 433 pairs of complexes, and the ratio of similarity, with different interface representations (separate Figures for each category are given in Figures 4 to 8 in Text S1). For each ABAC pair, the number of superimposed and similar elements is computed separately for each domain, and we compare the statistics on the homologous sides (A and A′) *versus* the non-homologous sides (B and C) of each complex. Each ABAC pair is thus represented by two points: one for complex AB and one for complex A′C. We previously checked that the sizes of the binding sites on A/A′ and B/C sides are roughly similar (see Figure 3 and Table 3 in Text S1), which is true, except for complexes of the M category, due to their protruding/interwound geometry as illustrated in Figure 2.

As expected, there is a positive correlation between the number of superimposed elements - defining the size of the overlap - on the A/A′ domains *versus* B/C domains, see Figures 3A, D and G, resulting from geometrical considerations. The number of superimposed elements is almost always lower on the B/C side than on the A/A′ side, for every interface representation. This is due to the fact that the structural superimposition is guided by domains A and A′, which favours better overlap on the A/A′ side, as illustrated in Figure 4. This bias introduced by the superimposition results in a mean ratio of overlap sizes equal to 1.3–1.8, depending on the interface representation: for 100 elements superimposed on the B/C side, there is an average of 130 to 180 elements on the A/A′ side (statistics for each pair category are presented in Table 4 in Text S1). Because of this effect alone, the number of similar elements on B/C sides is expected to be lower than on the A/A′ sides. It can be seen, in Figures 3B, E and H, that the number of similar elements on the B/C side is effectively lower. The mean numbers of similar elements for the five categories are given in Table 2. The mean ratio is around 2 for all-atom and coarse-grain representations and 3 for residues: there is, on average only one similar residue on the B/C side for 3 residues on the A/A′ side. Interestingly, the correlation between the similarity ratios, i.e., number of similar elements normalized by the number of superimposed elements (see Figures 3C, F and I) is lower. For example, the Pearson correlation coefficient between the numbers of similar atoms (see Figure 3B) is equal to 0.8, *versus* 0.4 between the corresponding similarity ratios (see Figure 3C). In other words, a greater similarity between A/A′ interfaces does not automatically correspond to a greater similarity between B/C interfaces. It thus seems that the low level of similarity in B/C domains is not only the result of the superimposition bias, but reflects a real sparsity of common binding determinants in different proteins that bind to similar partners. Indeed, some ABAC pairs with very similar common domains can exhibit very low similarity on the B/C sides. As an example, when complex 1m4u_BA (human bone morphogenetic protein-7 complexed with noggin) is compared with complex 1nys_DC (human activin A complexed with rat activin receptor) 11 out of 16 superimposed residues are similar for the A/A′ domain, and only 2 residues out of 9 for the B/C domain. Similar binding sites can thus bind two proteins that present a very restricted set of similar residues. To go further with this analysis, we computed similarity P-values as explained in the Materials and Methods section.

**Table 2:** Mean numbers of similar elements in different categories of ABAC pairs.

	All atoms			Coarse grain			C 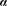		
Category^1^	N 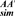 2	N 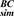 3	ratio^4^	N 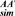	N 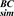	ratio	N 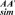	N 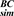	ratio
O	41	21	2.0	26	14	1.8	9	3	3
M	89	43	2.1	51	18	2.8	21	5	4.2
E	32	20	1.6	23	17	1.4	7	3	2.3
I	37	24	1.5	25	19	1.3	8	3	2.7
S	30	20	1.5	19	14	1.3	5	3	1.7
Total	44	23	1.9	28	16	1.8	9	3	3

1: Category of the pairs of complexes.

2: number of similar elements on domains A/A′.

3: number of similar elements on domains B/C.

4: ratio of N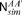 and N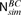.

### Significance of similarity

Similarity P-values, computed using a bootstrap procedure, are presented as histograms in Figure 5 for the ABAC pairs of category O.

A P-value equal to x% means that in x% of the randomly sampled interfaces, the number of similar elements is greater or equal to the number of similar elements in the real interface. Consequently, a high P-values indicates that the similarity has a high probability to occur by chance. Inversely, a very low P-value means that the similarity is significantly higher than expected with a random model. A value of 5% is classically used as the significance cut-off.

It is clear from Figures 5A and 5B that the distribution of similarity P-values is very different between A/A′ and B/C sides, with a bias toward low P-values on the A/A′ sides, and high P-values on the B/C sides. For A/A′ interfaces, we intuitively expect low P-values, indicating a significant similarity, since A and A′ domains belong to the same SCOP family and share a common ancestor. This is the case, see Figure 5A. What is less expected, is that the P-values for the B/C sides are rather high, indicating that the similarity between binding sites of the B and C domains is, most of the time, insignificant, see Figure 5B.

We note that the all-atom model (see Figure 5A) can however result in high P-values for A/A′ domains. This can be due to the background model used for bootstrapping, in which the atom type labels are randomly re-distributed among atom positions. In an all-atom representation, atoms of the same type appear as clusters, simply because they are part of the same amino acid. Such a random model is thus not optimal, because it neglects this aspect. Furthermore, with a distance cut-off equal to 3 Å to detect similar superimposed points, several atoms can be matched by the same point after superimposition. The result is an artificially high number of random similar points, and consequently, high P-values. Another source of error, with a probable significant impact, is the inherent sensitivity of the all-atom model to side chain flexibility. Since the same side chain, upon binding to multiple partners, might undergo different conformational changes, the all-atom model might under-estimate the real level of similarity. For these reasons we considered coarse-grain and C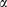 representations only in the following analysis.

As shown in Figures 5C and D, the coarse-grain representation overcomes the high P-value artifact on the A/A′ side. On the B/C histogram, however, a number of complexes still display high P-values, meaning that the similarity level is not significant compared to random. This holds true using a C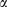 representation, see Figure 5E and F. We obtained similar results for other categories of ABAC pairs (Figures 4 to 7 in Text S1), although with more noisy results (less significant P-values on the A/A′ side) for the E, I and S categories, as expected due to the difficulty of the structural alignments for these categories.

### Evolutionarily conserved residues

We next considered the restricted set of evolutionarily conserved residues detected using the ConSurf database (as explained in the Materials and Methods section) and analyzed the interface similarity in this light. More precisely, we repeated the same analysis as for the C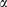 representation, but instead of considering five classes of residues, we labelled the residues by their conservation status, i.e., conserved or non-conserved. Then, we considered only the conserved residues at the interface, to see if they are co-localized with conserved residues after domain superimposition. As before, we computed separately the number of conserved residues superimposed on the A/A′ interfaces and the B/C interfaces, and the corresponding P-values. The P-value histograms follow the same trend as for binding site similarity: low P-values on the A/A′ side, but not on the B/C side, see Figures 8 and 9 in Text S1. Note that a considerable number of protein domains have no superimposed conserved residues in their binding sites, limiting the P-value analysis to a more restricted data set.

### Residues lying outside the overlap

As shown in Figure 6, interfaces are only partially overlapping after structural superimposition of A/A′ domains. We thus cannot exclude that some residues located outside the overlap play dominant roles in the binding. The correlation between the fraction of similar atoms and the fraction of atoms that are overlapping is weak but positive (see Figure 21 in Text S1). The fact that binding sites with a small fraction of similar atoms tend to have a small fraction of binding site overlap (meaning that a significant proportion of the binding site is excluded from the comparison) suggests that key binding determinants could indeed be missed.

## Discussion

In the same way that there is a limited number of protein folds, it is tempting to speculate that there is a limited number of protein-protein binding interfaces [62]. Since protein structures are made of recurrent local conformations, i.e., 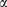-helices and 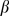-strands, protein-protein interfaces might be made by the assembly of recurrent binding modules. The present study was motivated by the search for such modules. Indeed, the fact that unrelated, dissimilar proteins are able to bind similar, homologous proteins suggests that common binding strategies might be re-used by different proteins. It is logical to look for generic binding modules in the promiscuous binding sites thus formed.

We were not however able to confirm this hypothesis. Starting from a discrete physico-chemical model, in which interfaces are described by points - be they atoms, pseudo-atoms or residues - belonging to five different classes, we found that, in most of the cases, the similarity between different proteins that bind to homologous partners is not greater than random (but the similarity between the homologous partners is significant, suggesting that the random model is appropriate). It thus seems that protein interfaces with no detectable similarity can nevertheless bind similar partners.

We should temper this result by noting that the energetic contribution of interfacial residues is uneven; some hot spot residues make major contribution, while other residues are unimportant. Unfortunately, energetic information - requiring extensive mutation analysis - is not available for our full data set, we thus approached this particularity in an indirect way. Although evolutionary conservation is a poor discriminant of hot spots [21], [22], it has been shown to improve the prediction when used in combination with other features [21]. Conserved residues do not translate into hot spots but might contain some information. We thus considered conserved residues at protein-protein interfaces, and assessed their co-localization in our complex pairs. This time, the criteria was not to know if superimposed residues are from the same physico-chemical class, but to know if they are both conserved during evolution, independently of their class. The rationale was to restrict the analysis to the subsets of conserved residues. The co-localization of conserved residues in different proteins that bind homologous partners was found to be largely insignificant. Further studies using *in silico* hot spot prediction methods could bring additional information.

Altogether, our results suggest the following picture for promiscuous protein-protein binding: similar, homologous proteins present binding sites with great similarity, *via* which they interact with diverse, dissimilar proteins. The binding interfaces of these dissimilar proteins exhibit different atomic/residue patterns, and their conserved residues are not co-localized. It thus suggests that different proteins use their own set of atoms/residues to perform the recognition, as illustrated in Figure 7A. There is also the possibility that atom groups interacting specifically with a single partner could play a dominant role, i.e., different partners use residues or group of residues that are outside the overlap between the two binding sites, see Figure 7B. The mechanism illustrated in Figure 7A is in agreement with the elegant work of Humphris and Kortemme, who have shown that multi-specific binding can be achieved by different mechanisms [33]. Using computational design to “optimize” the interfaces of promiscuous proteins, they observed two distinct patterns: (i) for half of the tested case, all partners shared key interactions; (ii) for the other half, each binding partner preferred its own set of wild-type residues in the common binding site. Some experimental studies of promiscuous proteins support this second pattern. For example, TRAF3 (Tumor Necrosis Factor Receptor-associated Factor) is able to bind two targets, CD40 and Lymphotoxin-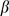 receptor, at the same interface, although they present motifs with distinct sequence and structure motifs for the binding [63]. Another example of promiscuous protein is protein kinase A, which is able to bind to different proteins using the same binding site. Entropy calculations suggest that the binding site of protein kinase A provides alternative contact points for the partner side chains [64]. In a recent study of BirA, a protein able to form a homodimer as well as heterodimer using the same binding site, hot spot residues were identified specifically for the homodimerization, but not for the heterodimerization [65]. This suggest that each complex forms using its own preferred and distinct interactions. This has also been observed for protein/ligand complexes. For example, different non-peptidic haptens have been shown to bind to the same site of an antibody, by forming different hydrogen bonds, dependent upon their particular chemistry and the availability of complementary antibody residues [66].

A last point to discuss is the existence of structural mimicry at interface. Protein mimicry is an intuitive concept, that has been successfully used in rational design [67]. Examples of protein interface mimicry - present in our data set - include several chymotrypsin inhibitors with various global folds (49 ABAC pairs), the viral protein M3 that mimics the binding site of chemokines for homodimerization (1 ABAC pair), and different subtilase inhibitors (3 ABAC pairs). Surprisingly, the similarity P-value analysis of these 53 pairs revealed that the physico-chemical similarity of the mimicking binding sites is not significant. However, their structural similarity is obvious, see Figure 2. This might indicate that the shape - not taken into account by our atomic or residue-based representations - is an important determinant for interface mimicry. Indeed, local surface comparison has been successfully used to retrieve chymotrypsin inhibitors [68].

The present study focused on promiscuous binding at the family level. The goal was to find the key determinants that allow unrelated proteins to bind to homologous partners. Our main conclusions are summarized below.

- Homologous proteins that bind different partners display different levels of structure similarity. Structural variation and residue insertion at the interfaces, as well as global structural variation, are seen in roughly one third of the ABAC pairs. This has to be taken into account in order to properly analyze the similarity of the binding sites.
- Structural mimicry at the interface of unrelated proteins that bind to homologous partners has been identified, but only for a limited number of ABAC pairs (53 out of 433 pairs), and an even more limited number of protein families (3 out of 105). Interface mimicry is thus probably not as generic as previously thought.
- Similarity between binding sites of unrelated proteins that bind to the same target is largely insignificant in terms of physico-chemical properties with similar spatial arrangement. That does not exclude the possibility that the same physico-chemical properties could be organized in a different manner between unrelated proteins.
- Conserved residues within the binding sites of unrelated proteins that bind to the same target are not co-localized.

We were not able to find evidence of convergent evolution. Our results support the hypothesis that promiscuous binding is rather achieved by alternative binding strategies for different partners.

## Materials and Methods

We exploited the data from the ABAC database (http://scoppi.biotec.tu-dresden.de/abac/) that contains protein-protein complexes organized in pairs [61]. As illustrated in Figure 4, ABAC pairs are formed by homologous proteins, A and A′, in interaction with non-homologous proteins B and C at equivalent binding sites. The SCOP classification [69] was used to ensure that A and A′ belong to the same family and B and C to different super-families. SCOP families gather proteins that have a clear evolutionary origin, measured by a sequence identity greater than 30%, or lower sequence identity, but very similar structure or function. At the superfamily level, proteins display low sequence identity, but structures and functions suggest that they are evolutionarily related. Proteins classified in different superfamilies are unrelated. Pairs with equivalent binding sites were selected after a two-stage procedure involving an assessment of interface residue overlap on A and A′ sequences and spatial overlap between A/B and A′/C interfaces measured by the angle between the center of mass of A/A′, and the center of mass of the interfacial region of B and C [61].

### Data set

PDB files of protein-protein complexes were retrieved from the PQS database [70]. Starting from a non-redundant list of ABAC pairs with only one instance per SCOP family combination, we selected pairs that fulfilled two criteria: (i) the two partners are from different chains, *i.e.*, we do not consider intra-chain interactions, (ii) SCOP domains spanning several protein chains involved in the binding site are excluded from the analysis for computational simplicity. We also removed complexes with missing atomic coordinates at the binding site, and pairs with very low overlap between the binding sites resulting in no superimposed atoms on the B/C side. Details concerning the minimum overlap size in the data set are given in Table 5 in Text S1. The final data set comprises 433 ABAC pairs. These 433 pairs were further classified into 5 categories, based on a visual assessment of the quality of the superimposition between A and A′ domains, particularly at the interfaces:

- O (optimal class): there is a good superimposition between A and A′, 246 pairs,
- M (mimicry): same as O, but in addition, domains B and C are an example of structural mimicry at the binding site, 53 pairs,
- E (ensemble conformation): domains A and A′ display alternate conformations at the interface, 63 pairs,
- I (insertion/deletion): domains A and A′ differ by an insertion/deletion at the interface, 21 pairs,
- S (superimposition problem): global superimposition between A and A′ is poor, due to structural variability between A and A′, 50 pairs.

For the category M, the geometry of the main chain of B and C domains in the binding site was taken into account. Globally, O and M categories correspond to smaller rmsd between A and A′ domains, and smaller irmsd (rmsd between interfacial residues) compared to category S; categories E and I are intermediate; and categories overlap in terms of rmsd values, see Figure 2 in Text S1. Note that rmsd and irmsd are *average* values of structural deviation, hence they only reflect global tendencies; furthermore, they depend on the extent of the structural alignments. Also, irmsd computation does not take into account insertion of residues, because they are unaligned. Structural mimicry of B and C domains cannot be detected using rmsd, since domains B and C are unrelated and hence not superimposable. The classification thus ultimately results from a careful visual examination that takes into account all these parameters.

Our data set is non-redundant in the sense that every SCOP family combination is unique. However, the ABAC pairs are not independent, since the same SCOP family can be shared by several pairs. For example, the SCOP family 49504 (Plastocyanin/azurin-like) is shared by the A/A′ domains of two ABAC pairs:

- PDB structure 1mg2, chains OP (amicyanin of *Paracoccus denitrificans* complexed with cytochrome c551) *versus* PDB structure 1gr7, chains BC (dimer of azurins of *Pseudomonas aeruginosa*),
- PDB structure 7pcy, chains AC (dimer of plastocyanins of *Enteromorpha prolifera*) *versus* PDB structure 1mda, chains AM (amicyanin of *Paracoccus denitrificans* complexed with the light chain of methylamine dehydrogenase).

Overall, 68 SCOP families are present in only one ABAC pair if we consider their A/A′ domains, and the most abundant family - family 52592, G proteins - is represented in 130 pairs. This probably indicates both the capacity of some particular families for promiscuous binding at the family level, but may also reflect the bias of structures deposited in the PDB toward proteins with biomedical interest. The number of distinct SCOP families, for A/A′ domains and B/C domains are reported in Table 1, for each category of ABAC pairs. It can be seen that the number of different SCOP families in A/A′ domains is 105 for the full data set. This apparent redundancy is not a limitation in our context, since we consider the similarity between *pairs* of complexes. In particular, considering ABAC pairs with unique SCOP domain combinations is enough to explore how different B/C domains interact with similar A/A′ domains.

### Comparison of binding sites

Interfacial atoms were detected by applying a cut-off of 5 Å between heavy atoms from interacting chains, as in the SCOPPI database [71], [72]. Residues were considered to be part of the binding site if they had at least one interfacial atom.

Atoms were classified into five groups adapted from those proposed by Mintseris and Weng [73] (see Figure 1 in Text S1). These groups were determined by an optimization procedure, so as to maximize the mutual information of the pairwise matrix of atomic contacts at protein-protein interfaces. Although they have been determined by statistical optimization, they are in excellent agreement with biochemical criteria and roughly make the distinction between positively charged/negatively charged/polar/non-polar and hydrophobic groups of atoms.

As in [61], homologous partners of the ABAC pairs, *i.e.*, domains A and A′, were superimposed using Multiprot [74]. After structural superimposition, interfacial atoms from A (resp. B) were considered as *superimposed* if there was an interfacial atom from A′ (resp. C) less than 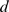 Å away, and *similar* if both atoms were from the same group. Cutoff 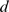 was set to 3 Å, as in [61]. Note that this cut-off is used to compute the number of similar atoms between two binding sites after superimposition, and should not be confused with the cut-off equal to 5 Å that is used to detect atoms that are part of the interface.

Binding site similarity was also quantified on a per-residue basis, by representing each residue by its C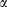. In addition, we considered an intermediate coarse-grain model introduced by Zacharias [75], in which residues - except GLY - are modeled by two or three pseudo-atoms: the C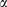, and one side-chain pseudo-atom (residues ALA, SER, THR, VAL, LEU, ILE, ASN, ASP and CYS) or two side-chain pseudo-atoms (residues PHE, MET, PRO, TRP, HIS, TYR, GLN, GLU, LYS, ARG). Residues and pseudo-atoms were clustered into five groups, deduced from the atom groups (see Tables 1 and 2 in Text S1).

In order to take into account the fact that residues are described by a reduced number of points using these simplified representations, the cut-off to detect similar points after complex superimposition was empirically set to 4 Å for the C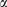 and the coarse-grain representations.

### Significance of binding site similarity

The significance of the similarity between binding sites was assessed by bootstrapping. The principle is to generate random binding sites by randomly re-assigning the atom types in the overlapping interfaces. The advantage of this re-sampling is that the sizes of the compared objects are preserved. The procedure was repeated 500 times in order to obtain the distribution of the number of similar atoms (or pseudo-atoms or residues) between two binding sites that can be expected with a random model. The extent of the observed similarity could then be assessed by computing the corresponding P-value, 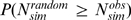, where 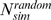 and 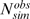 denote respectively the number of similar atoms obtained between random binding sites, and observed between real binding sites. For each ABAC pair, we thus computed four P-values: one for each of A, A′, B and C binding sites.

### Evolutionarily conserved residues

Evolutionarily conserved residues were detected using the ConSurf database [76]. This database contains pre-calculated conservation scores, obtained after multiple alignment of homologous sequences using an empirical Bayesian algorithm [77]. For each residue of a protein, a normalized conservation score is assigned. Residues with normalized scores lower than -1 were considered as evolutionarily conserved. In some cases, when the number of homologous sequences is too low, the conservation scores were not available. In such cases, all residues were considered as unconserved.

During the comparison of binding sites, 131 comparisons out of 433 involved a binding site with no conserved residues when considering A/A′ domains, and 178 out of 433 when considering B/C domains. The analysis of evolutionarily conserved residues is thus inherently based on a smaller data set.

## Supporting Information

Text S1 Supporting Figures and Tables.
(1.23 MB PDF)
