# Supplementary material for: Beauty Is in the Eye of the Beholder: Proteins Can Recognize Binding Sites of Homologous Proteins in More than One Way
Source: PLoS Comput Biol. 2010 Jun 17;6(6):e1000821. doi: 10.1371/journal.pcbi.1000821 (PMC2887470; doi:10.1371/journal.pcbi.1000821)
Supplement: Text S1 — Supporting Figures and Tables. (1.23 MB PDF) [file pcbi.1000821.s001.pdf]

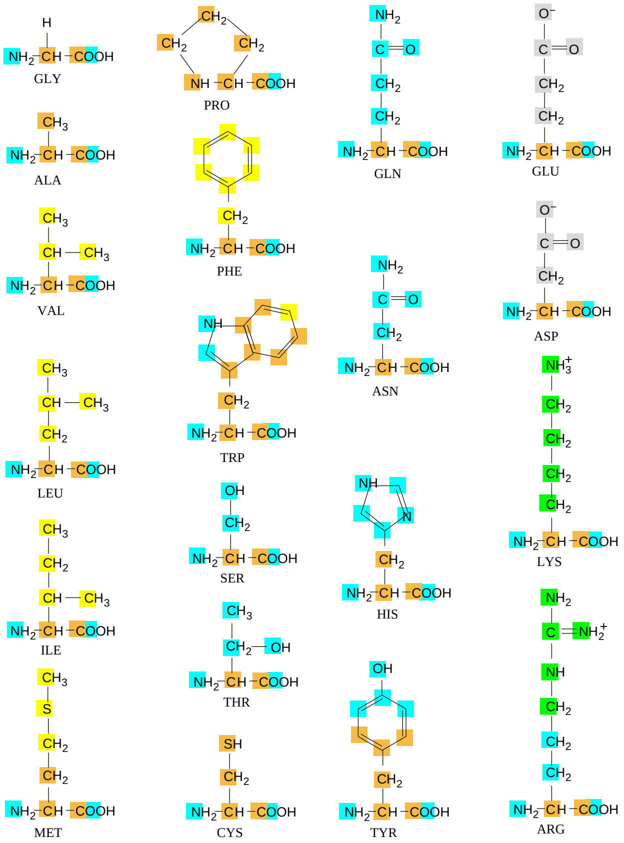

Figure 1: **The five atom groups used in the present study.** We took the same grouping as the one obtained by Mintseris and Weng on a data set of 327 protein interfaces [2], and modified it so as to have identical grouping for main-chain atoms. The five groups roughly represent: yellow: hydrophobic side chains, green: positively charged side-chains, blue: side chains of polar residues, polar portion of TYR and TRP, main-chains Hbond donors/acceptors, gray: negatively charged side-chains, orange: ALA, CYS, other main-chain atoms and non-polar portions of TYR and TRP.

1	VAL_SC, LEU_SC, ILE_SC, PHE_SC1, PHE_SC2, MET_SC2
2	LYS_SC1,LYS_SC2,ARG_SC2
3	All Cα, ALA_SC, SER_SC,THR_SC, GLN_SC1,GLN_SC2, ASN_SC1,ASN_SC2, HIS_SC2, TYR_SC2,ARG_SC1
4	GLU_SC1, GLU_SC2, ASP_SC1, ASP_SC2
5	MET_SC1, PRO_SC1, PRO_SC2, TRP_SC1, TRP_SC2, CYS_SC, HIS_SC1, TYR_SC1

Table 1: Grouping for coarse-grain representation. Here, _SC refers to side-chain pseudo-atoms.

1	VAL, LEU, ILE, MET,PHE
2	LYS, ARG
3	ALA, SER, THR, GLN, ASN, HIS, TYR
4	GLU, ASP
5	GLY, PRO, TRP, CYS

Table 2: Grouping for Cα representation.

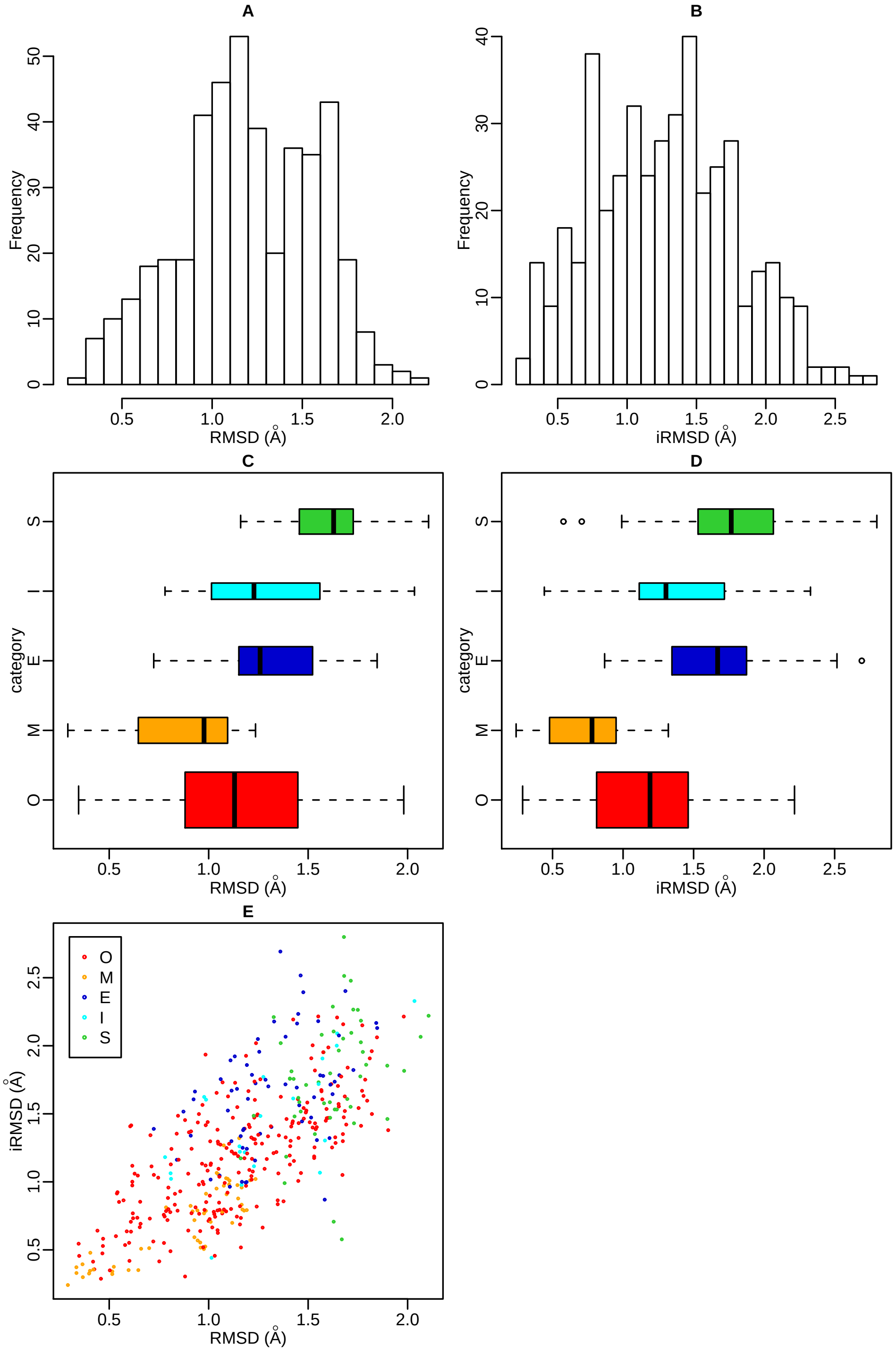

Figure 2: **Features of the five categories of ABAC pairs.** A: distribution of rmsd between A and A' domains, computed using Multiprot for all pairs, B: distributions of irmsd, the rmsd between interfacial residues of A and A', computed from the residue pairs given by Multiprot, for all pairs, C: distribution of rmsd between A and A' domains for each category, D: distribution of irmsd between A and A' interfaces, for each category, E: biplot of rmsd *versus* irmsd. The width of the boxplots in C and D indicates the number of ABAC pairs in each category.

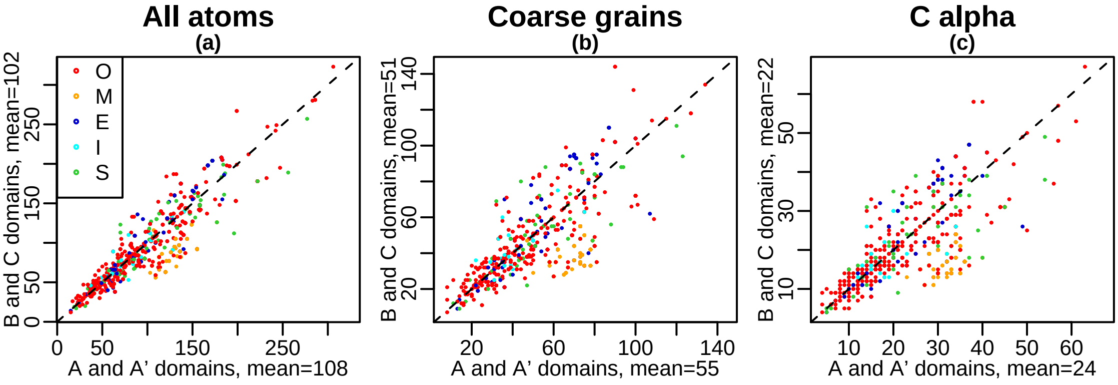

Figure 3: **Size of protein-protein interfaces.** (a): all-atom representations, (b): coarse-grain representations, (c): Cα representations.

Table 3: Mean sizes of protein-protein interfaces in different categories of ABAC pairs.

	All atoms			Coarse grain			Cα		
Category[1]	$N^{AA'}$[2]	N^{BC}[3]	ratio[4]	$N^{AA'}$	N^{BC}	ratio	$N^{AA'}$	N^{BC}	ratio
O	101	98	1.0	50	48	1.0	22	21	1.0
M	128	87	1.3	68	39	1.7	31	17	1.8
E	117	124	0.9	59	66	0.9	25	28	0.9
I	100	101	1.0	51	52	1.0	22	23	1.0
S	117	113	1.0	57	58	1.0	25	25	1.0
Total (433)	108	102	1.1	55	51	1.1	24	22	1.1

1: Category of the pairs of complexes, 2: number of interfacial elements on domains A/A', 3: number of interfacial elements on domains B/C, 4: ratio of $N^{AA'}$ and N^{BC}.

Table 4: Mean numbers of superimposed elements in different categories of ABAC pairs.

	All atoms			Coarse grain			Cα		
Category[1]	$N^{AA'}$[2]	N^{BC}[3]	ratio[4]	$N^{AA'}$	N^{BC}	ratio	$N^{AA'}$	N^{BC}	ratio
O	56	43	1.3	31	23	1.3	14	8	1.8
M	105	62	1.7	57	27	2.1	26	11	2.4
E	49	43	1.1	30	27	1.1	12	8	1.4
I	52	45	1.2	32	28	1.1	13	9	1.4
S	50	47	1.1	28	24	1.2	12	8	1.5
Total (433)	60	45	1.3	34	24	1.4	15	8	1.8

1: Category of the pairs of complexes, 2: number of superimposed elements on domains A/A', 3: number of superimposed elements on domains B/C, 4: ratio of $N^{AA'}$ and N^{BC}.

Table 5: Minimal numbers of superimposed elements in ABAC pairs.

Category[1]	$\min^{AA'}$	$\min^{BC}$	$q_{5\%}^{AA'}$	$q_{5\%}^{BC}$
	All atoms			
O	10	4	20	12
M	68	33	86	47
E	4	2	9	11
I	19	7	21	10
S	8	10	18	13
Total	4	2	20	12
	Coarse grains			
O	8	4	13	8
M	31	15	48	17
E	2	4	10	8
I	12	7	14	8
S	7	4	14	8
Total	2	4	13	8
	Cα			
O	4	1	6	2
M	15	4	21	6
E	1	2	4	2
I	5	1	5	2
S	2	1	4	2
Total	1	1	6	2

1: Category of the pairs of complexes, min: minimum number of superimposed elements, $q_{5\%}$: 5% quantile of the distribution; a quantile equal to 10 means that 5% of the interface have less than 10 superimposed points (and consequently, 95% of the data is above this limit).

Table 6: Mean fraction of similar elements in different categories of ABAC pairs.

	All atoms			Coarse grain			Cα		
Category[1]	$F^{AA'2}$	F^{BC3}	ratio[4]	$F^{AA'}$	F^{BC}	ratio	$F^{AA'}$	F^{BC}	ratio
O	0.7	0.5	1.4	0.8	0.6	1.3	0.6	0.4	1.5
M	0.8	0.7	1.5	0.9	0.7	1.3	0.8	0.4	2.0
E	0.6	0.4	1.5	0.8	0.6	1.3	0.5	0.4	1.2
I	0.7	0.5	1.4	0.7	0.6	1.2	0.5	0.3	1.7
S	0.6	0.4	1.2	0.7	0.6	1.2	0.4	0.3	1.3
Total (441)	0.7	0.5	1.4	0.8	0.6	1.3	0.6	0.3	1.5

1: Category of the pairs of complexes, 2: fraction of similar elements on domains A/A', 3: fraction of similar elements on domains B/C, 4: ratio of $F^{AA'}$ and F^{BC}.

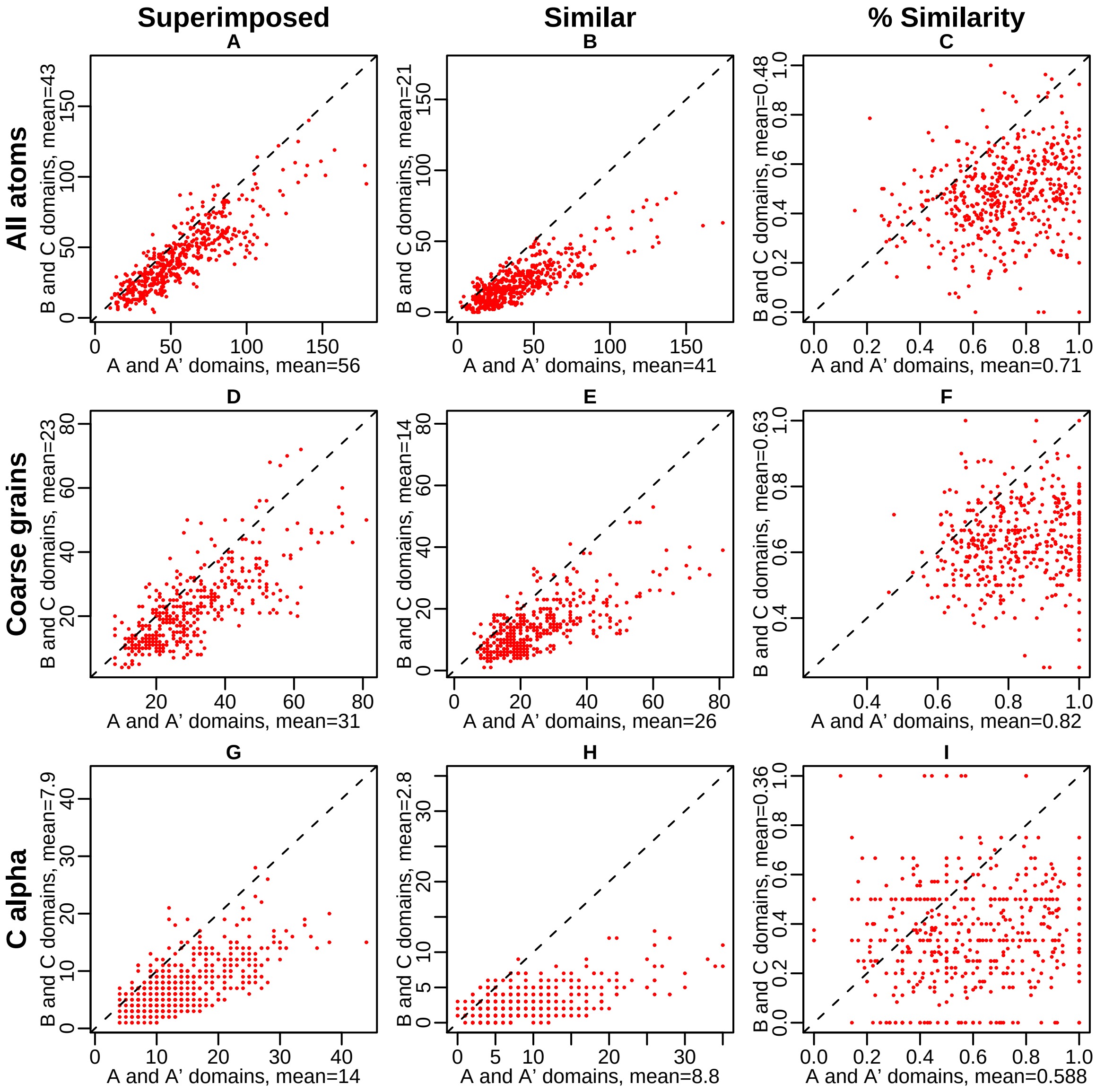

Figure 4: **Similarity at protein-protein interfaces in ABAC pairs of the category O.** First row: all-atom representations, second row: coarse-grain representations, third row: Cα representations. First column: number of superimposed elements on A/A' *versus* B/C side, second column: number of similar elements on A/A' *versus* B/C side, third column: fraction of similar elements on A/A' *versus* B/C side.

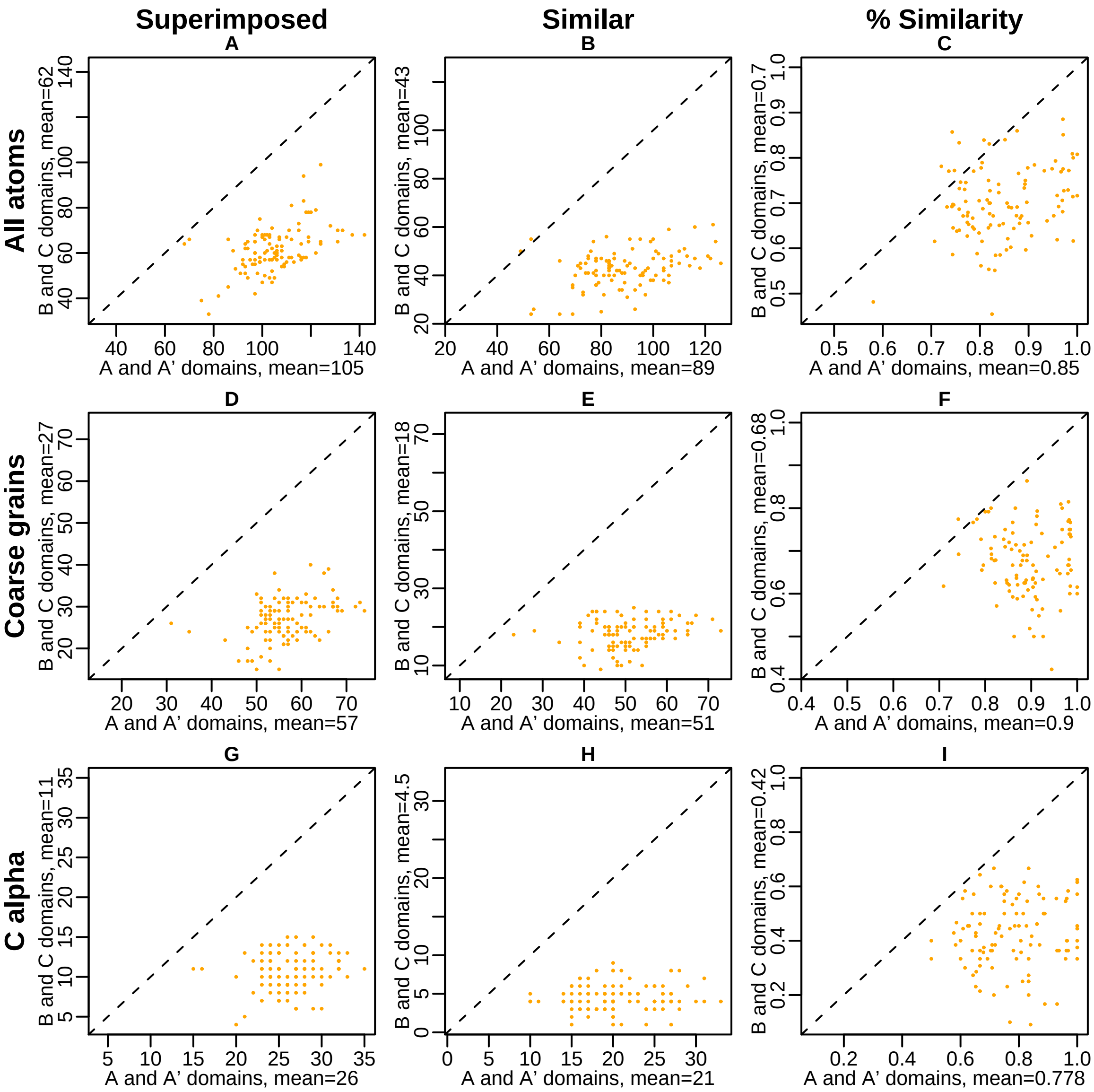

Figure 5: **Similarity at protein-protein interfaces in ABAC pairs of the category M.** See Figure 4 for details.

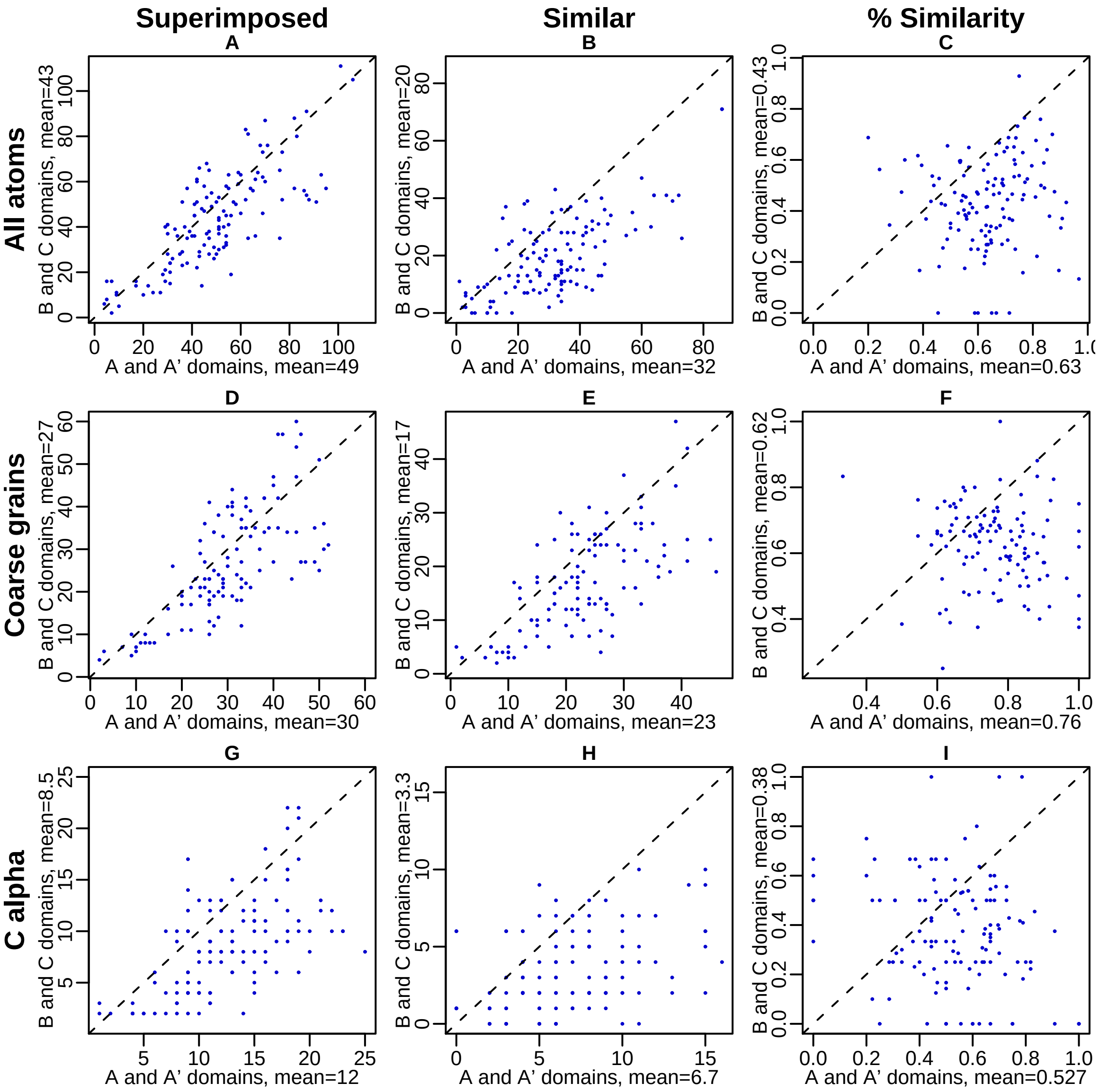

Figure 6: **Similarity at protein-protein interfaces in ABAC pairs of the category E.** See Figure 4 for details.

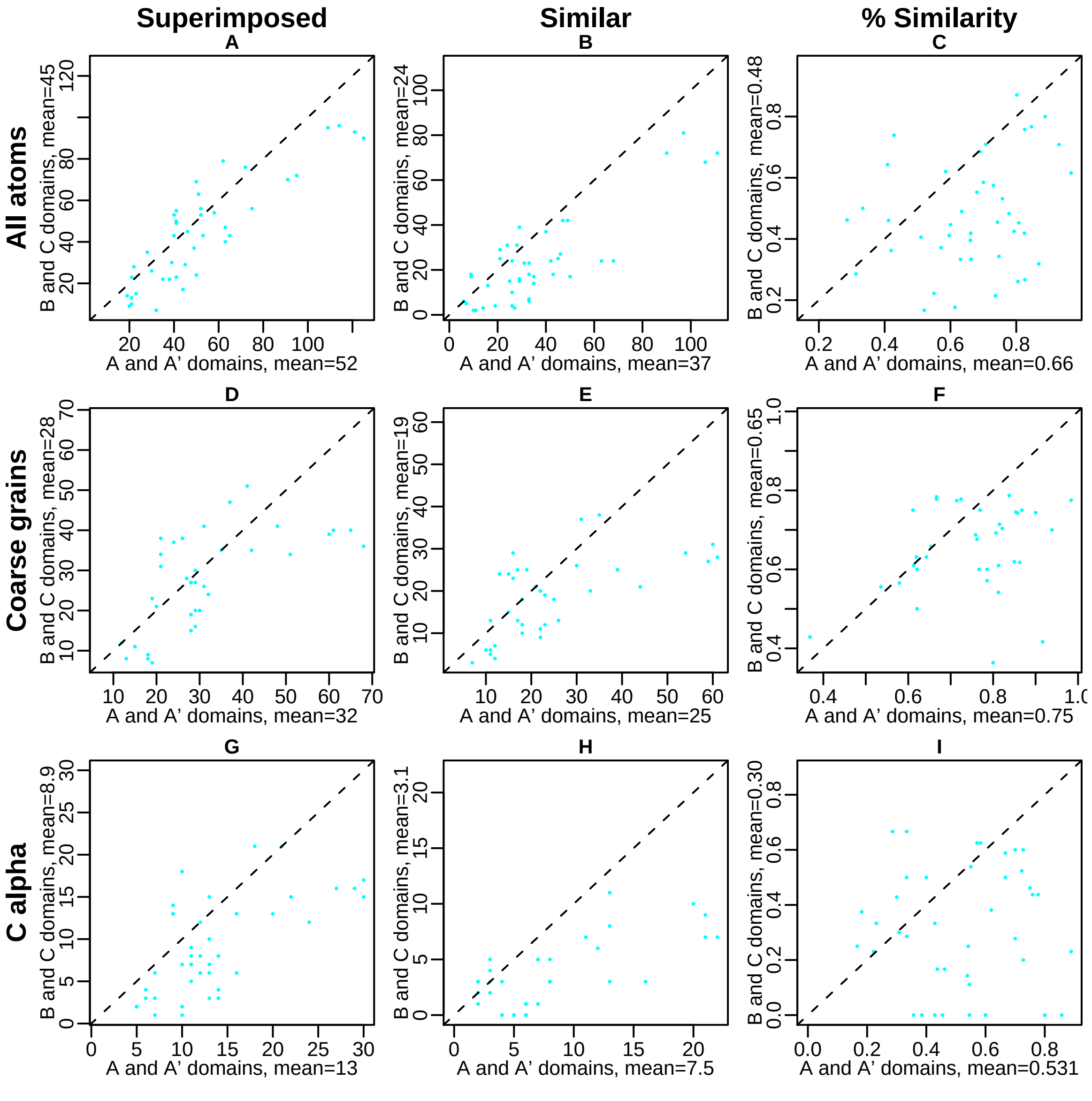

Figure 7: **Similarity at protein-protein interfaces in ABAC pairs of the category I.** See Figure 4 for details.

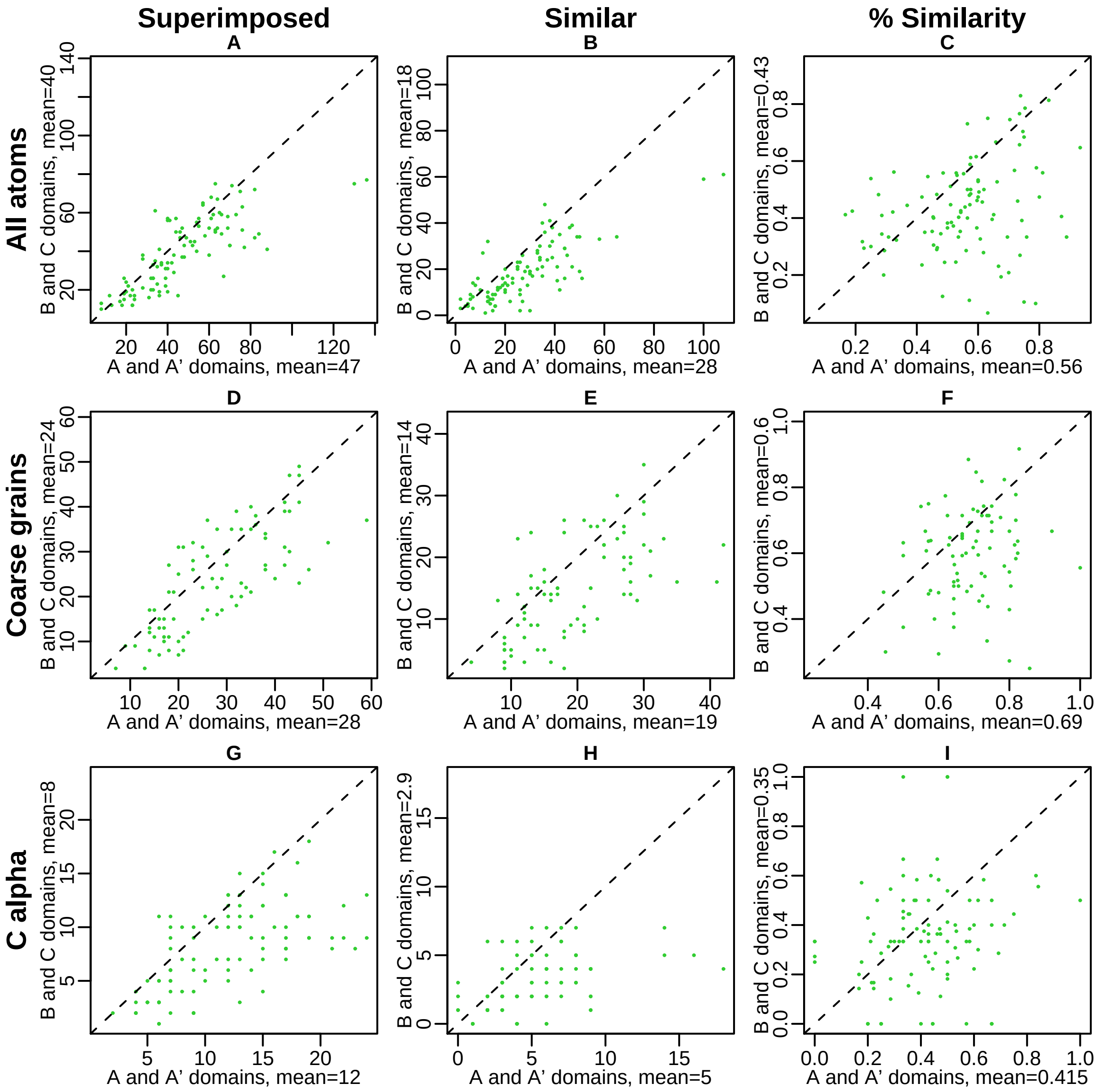

Figure 8: **Similarity at protein-protein interfaces in ABAC pairs of the category S.** See Figure 4 for details.

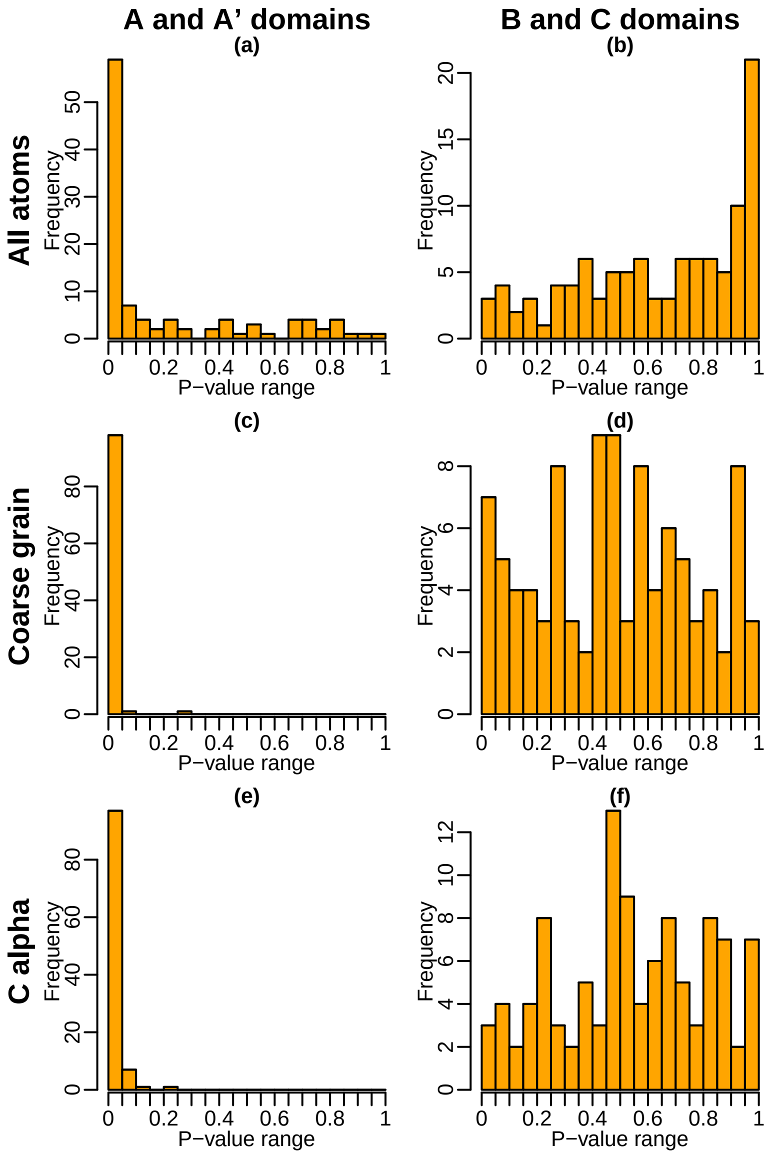

Figure 9: **Distribution of similarity P-value at protein-protein interfaces of ABAC pairs of the category M.** First row: all-atom representations, second row: coarse-grain representations, third row: Cα representations, first column: P-values of the A/A' domains, second column: P-values of the B/C domains. White bars correspond to a number of similar elements equal to zero, which, by definition, yields a P-value equal to 1, since the random model cannot give a number of similar residues lower than zero (see following figures).

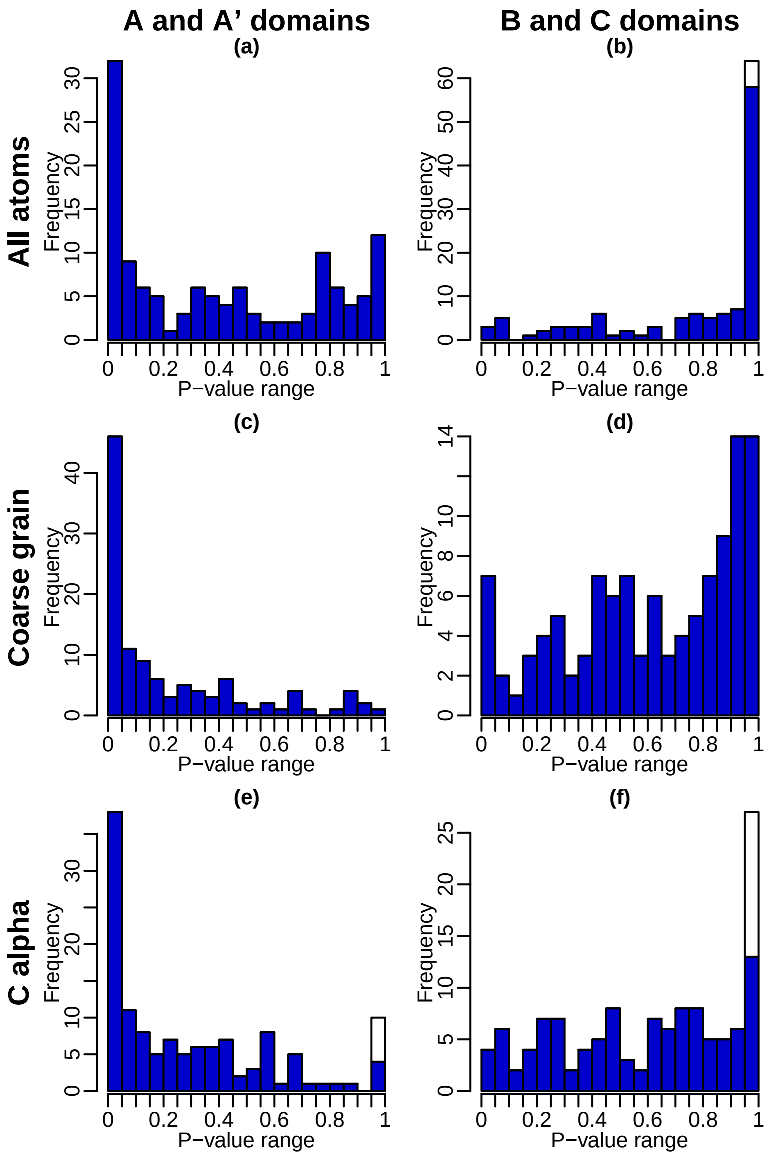

Figure 10: **Distribution of similarity P-value at protein-protein interfaces of ABAC pairs of the category E.** See Figure 18 for details.

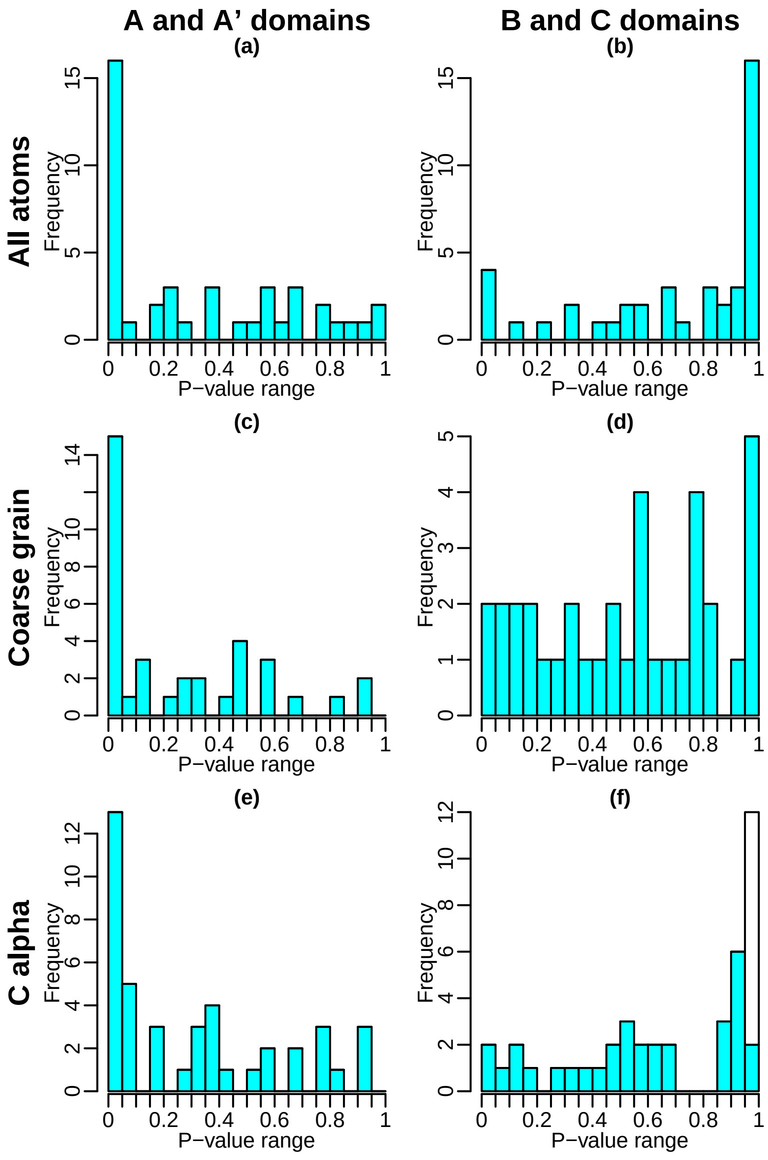

Figure 11: **Distribution of similarity P-value at protein-protein interfaces of ABAC pairs of the category I.** See Figure 18 for details.

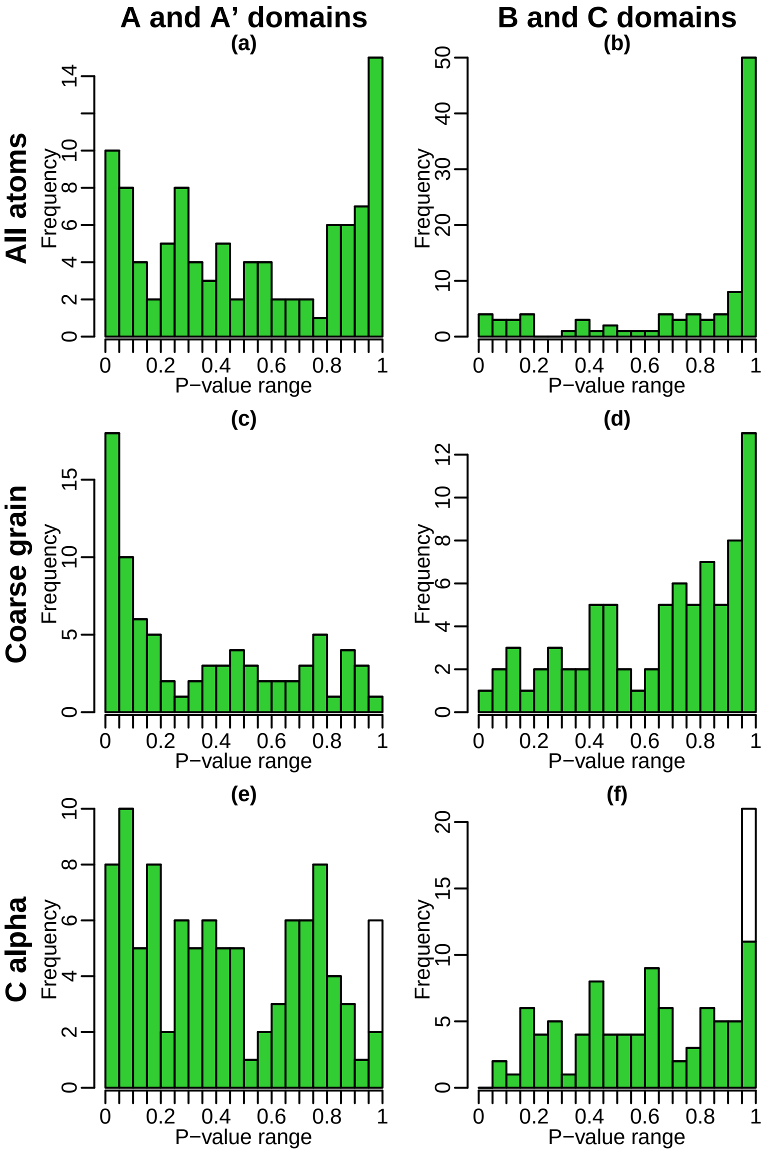

Figure 12: **Distribution of similarity P-value at protein-protein interfaces of ABAC pairs of the category S.** See Figure 18 for details.

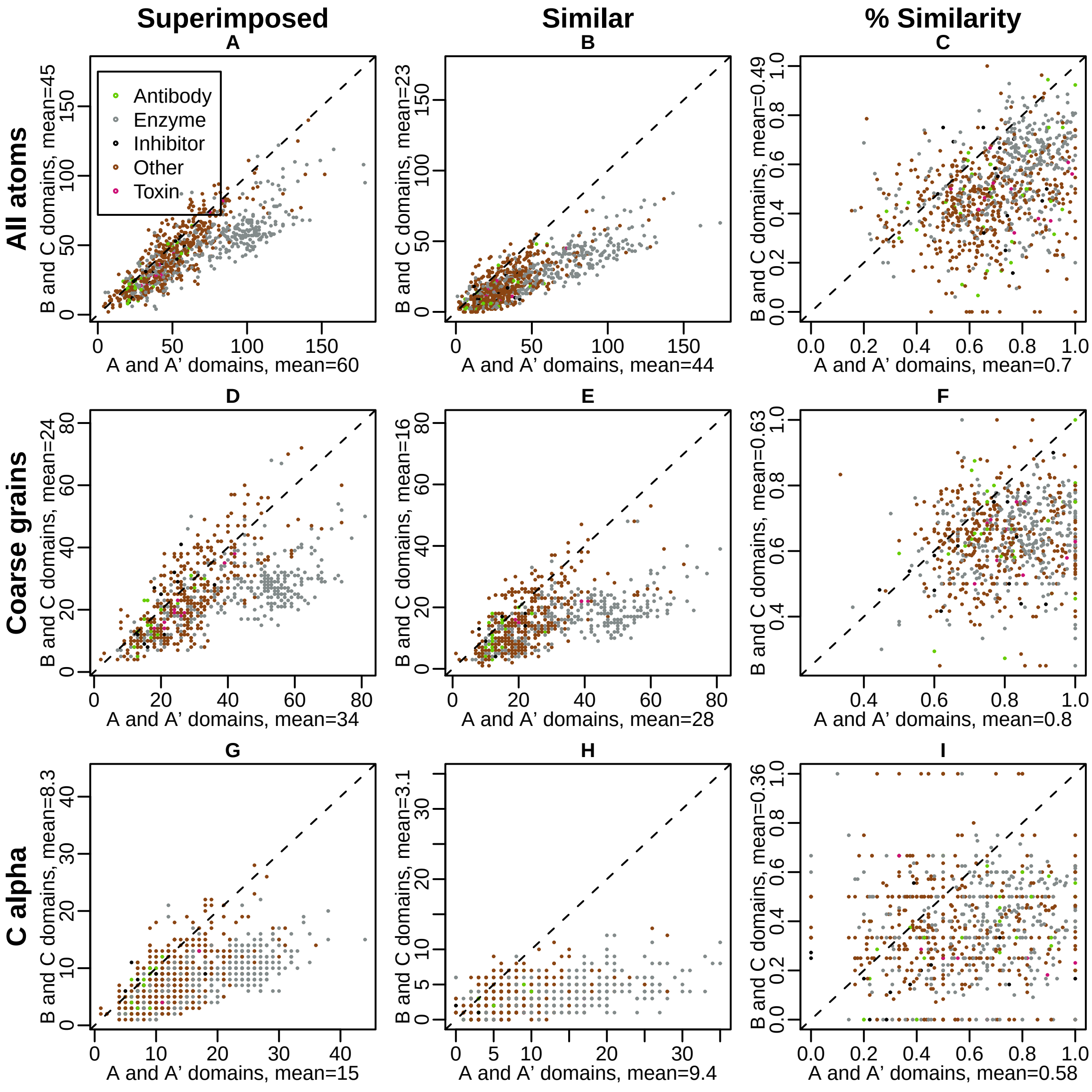

Figure 13: **Similarity at protein-protein interfaces in ABAC pairs.** Pairs of complexes are classified into five functionnal categories according to the SCOP family of the A/A' domains: Antibody (16 pairs), Enzyme (185 pairs), Inhibitor (8 pairs), Toxin (6 pairs) and Other (218 pairs).

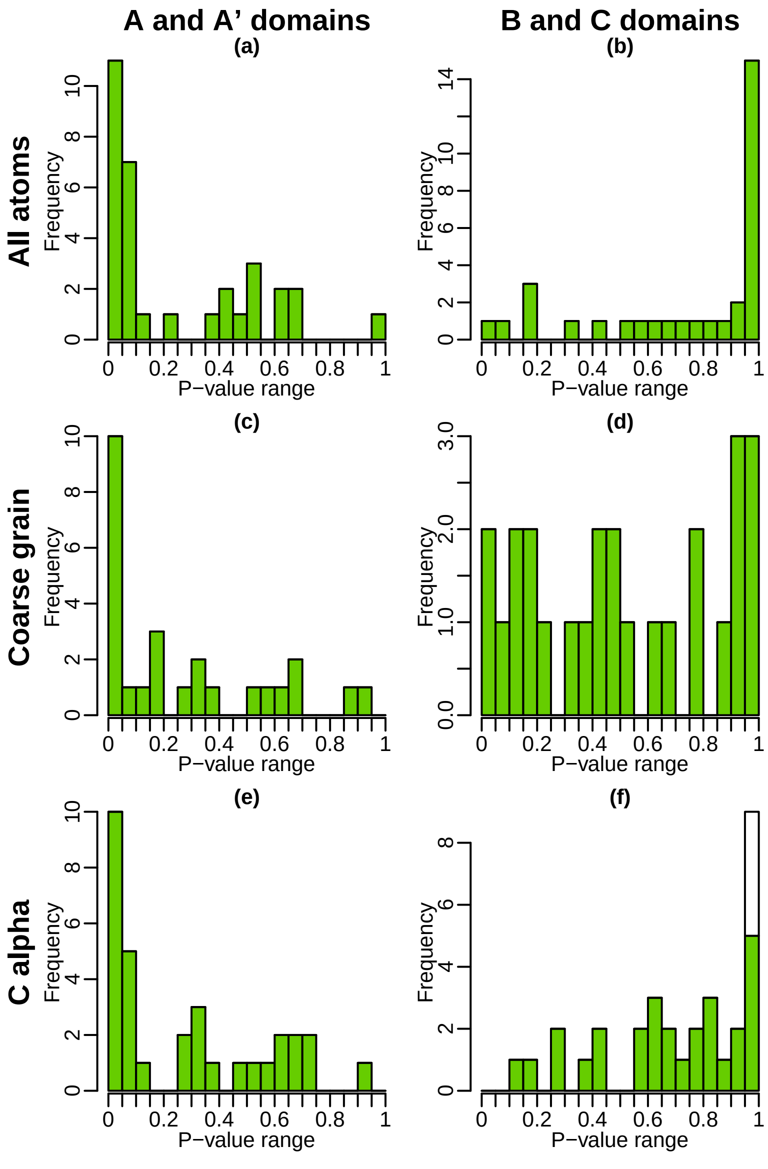

Figure 14: **Distribution of similarity P-value at protein-protein interfaces of ABAC pairs of the category Antibody.**

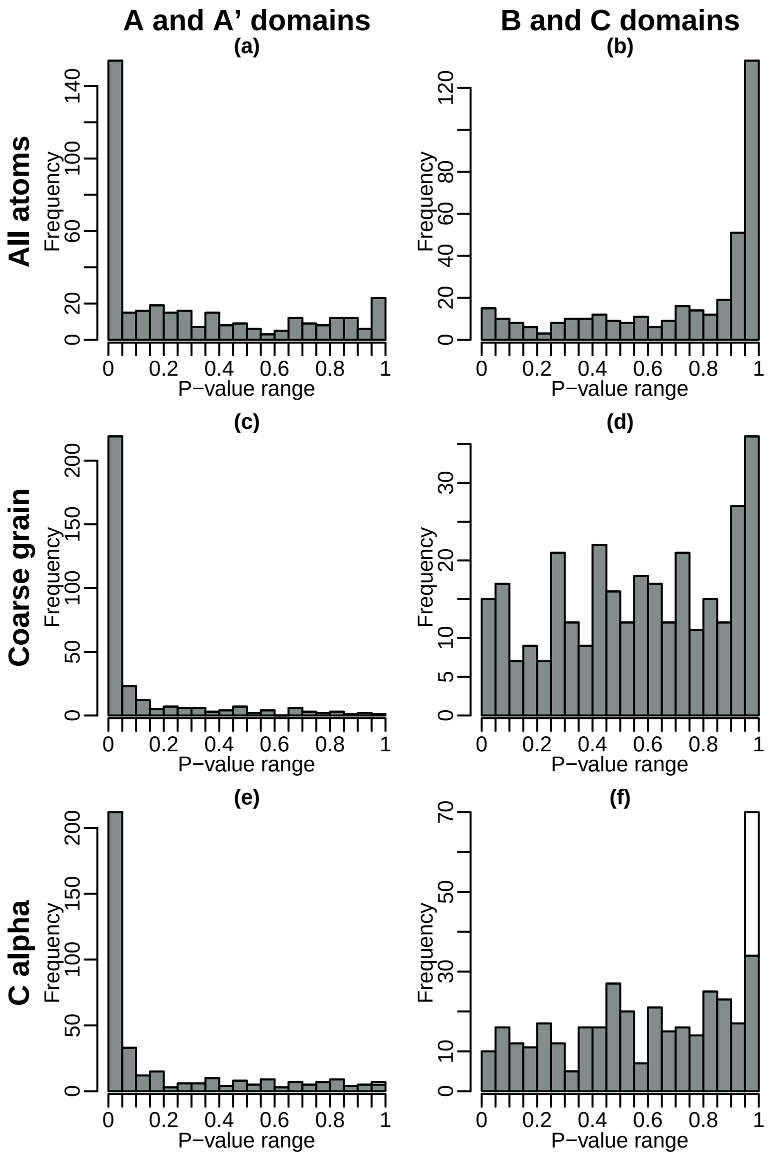

Figure 15: **Distribution of similarity P-value at protein-protein interfaces of ABAC pairs of the category Enzyme.**

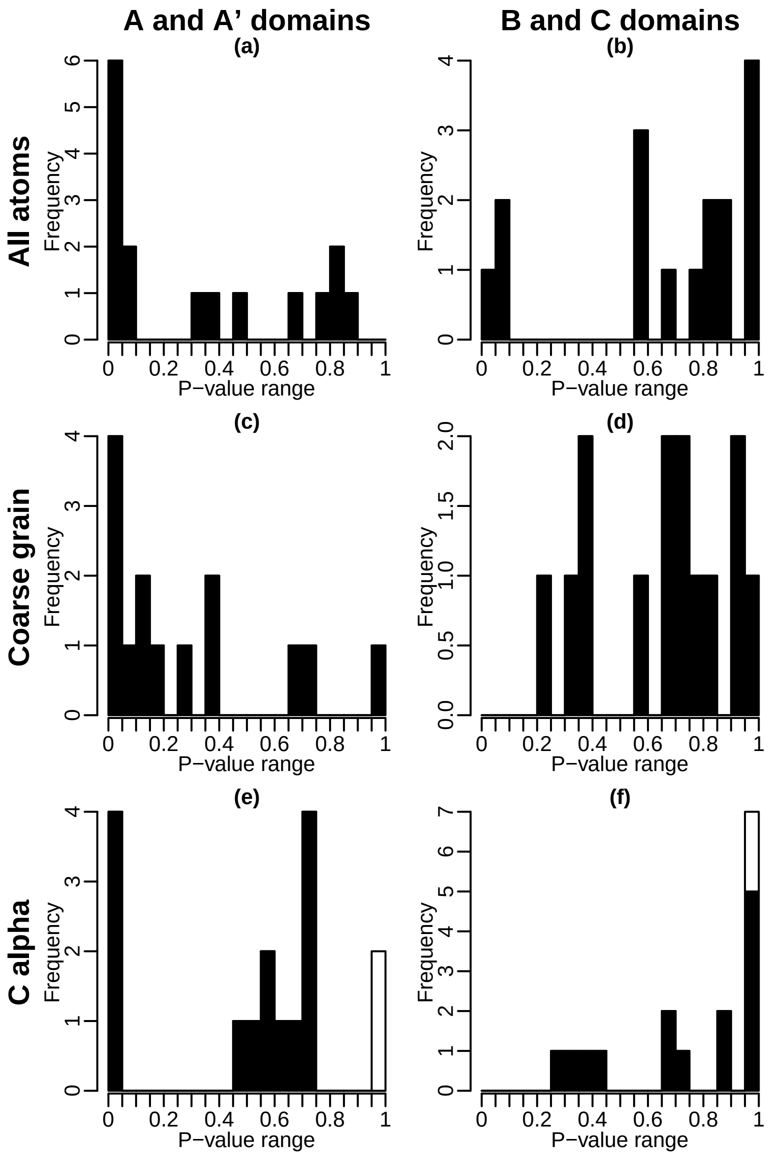

Figure 16: **Distribution of similarity P-value at protein-protein interfaces of ABAC pairs of the category Inhibitor.**

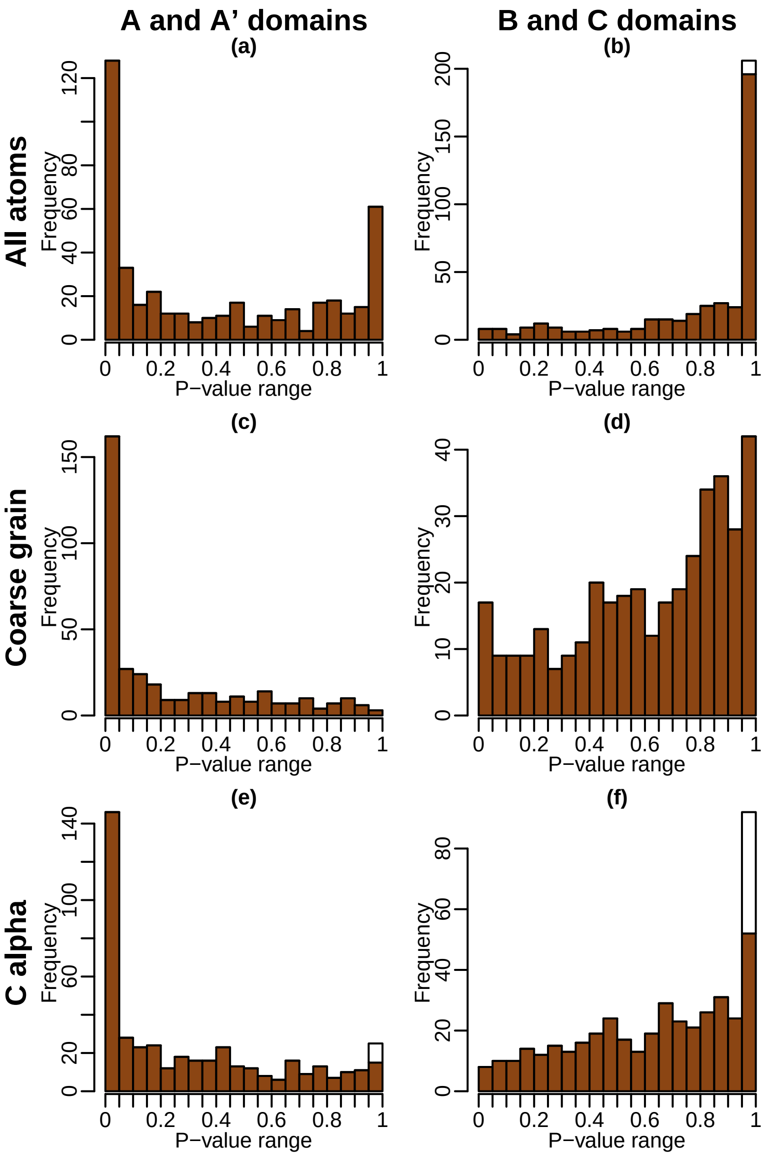

Figure 17: **Distribution of similarity P-value at protein-protein interfaces of ABAC pairs of the category Other.**

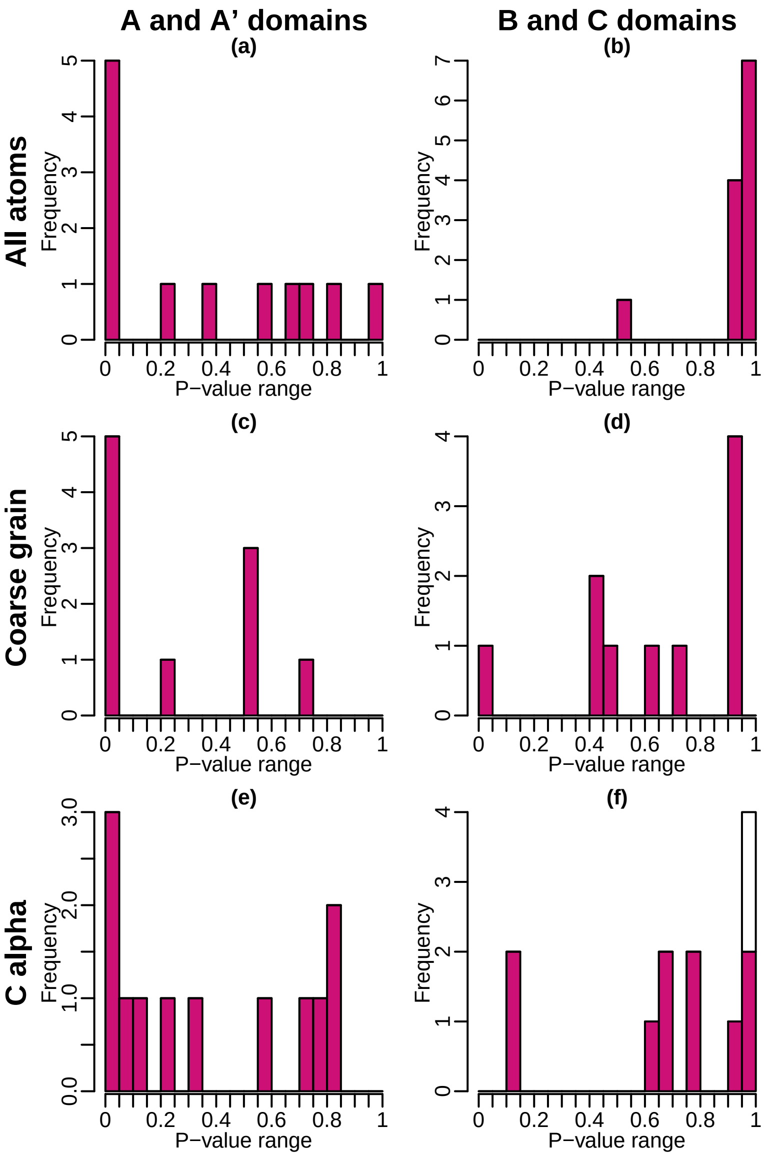

Figure 18: **Distribution of similarity P-value at protein-protein interfaces of ABAC pairs of the category Toxin.**

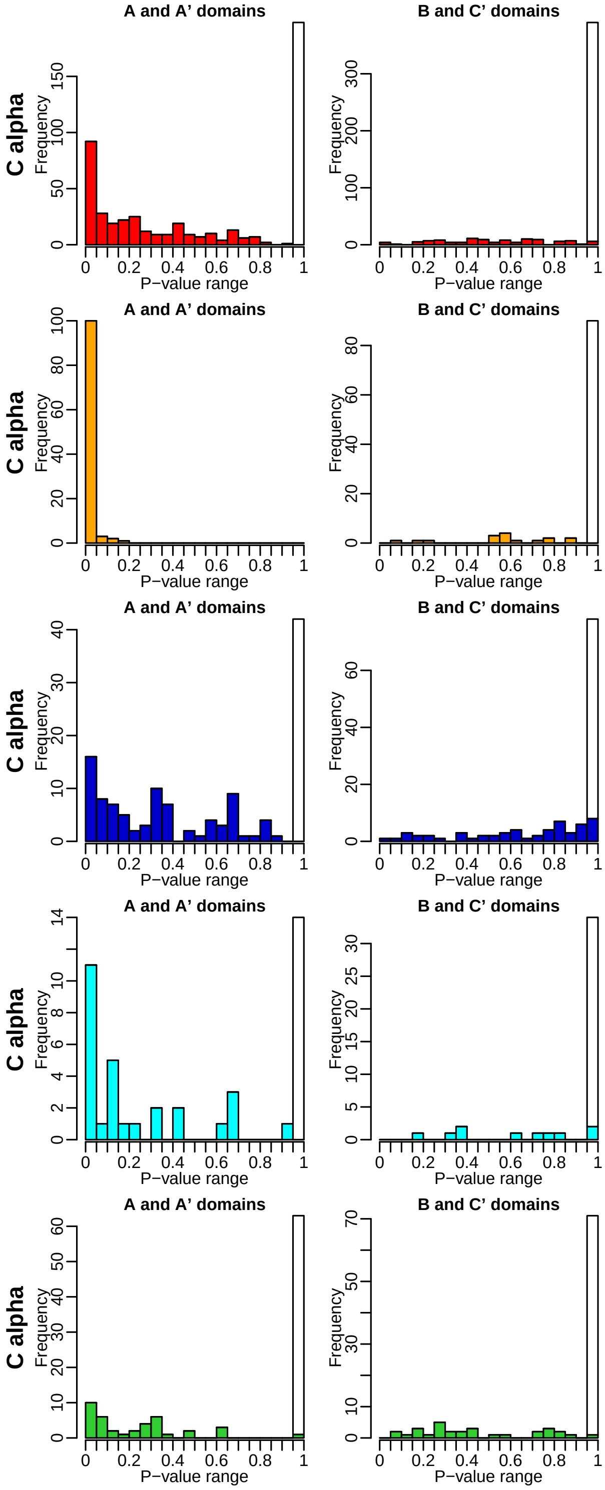

Figure 19: **Distribution of P-values for the co-localization of conserved residues (residues with ConSurf score < -1) at protein-protein interfaces of ABAC pairs of all categories.** Scheme color is the same as in Figure 4. White bars correspond to a number of conserved residues equal to zero.

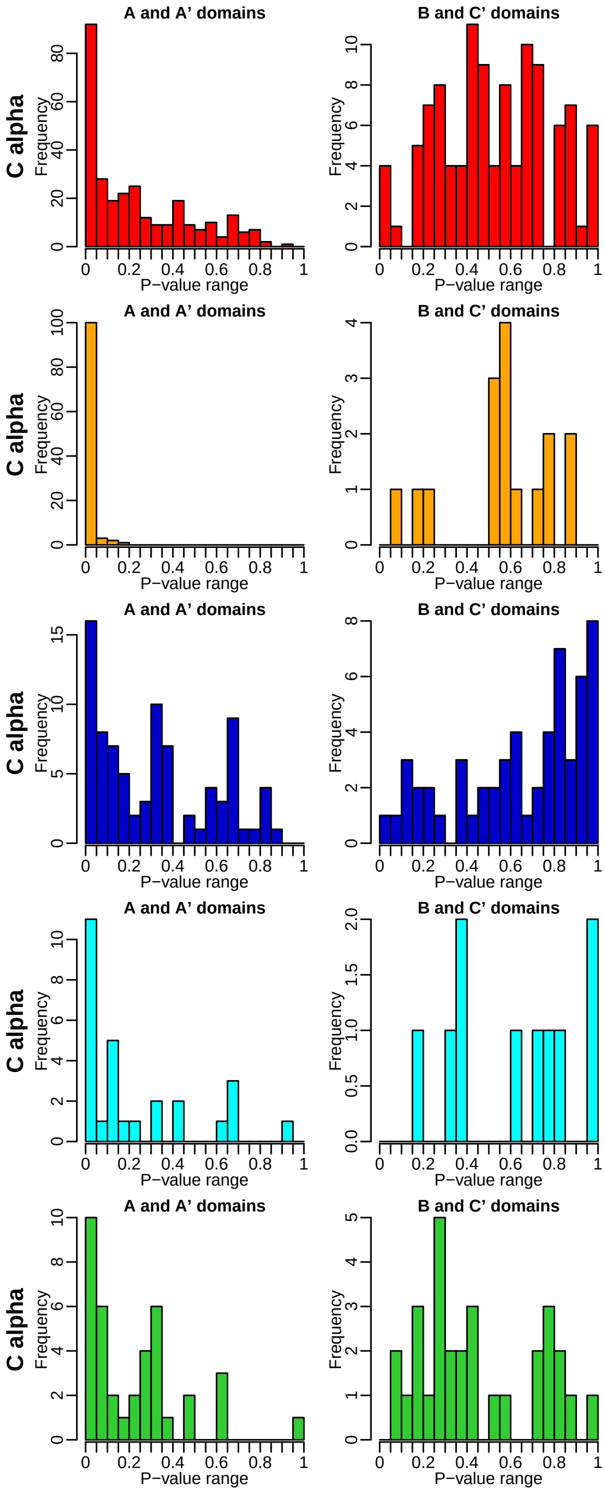

Figure 20: **Distribution of P-values for the co-localization of conserved residues (residues with ConSurf score < -1) at protein-protein interfaces of ABAC pairs of all categories.** Same data as Figure 19, but domains with no conserved residues are ignored.

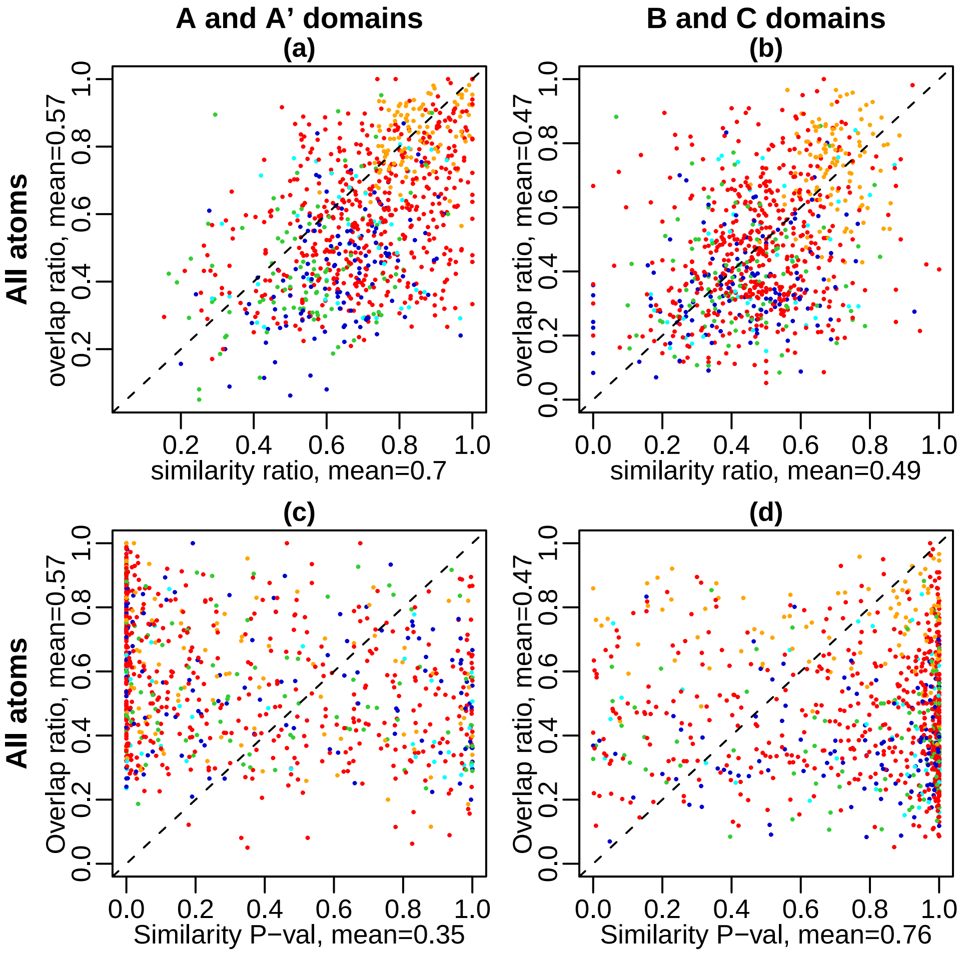

Figure 21: **Overlap *versus* similarity of interfaces in ABAC pairs.** Overlap ratio is the number of superimposed atoms over atoms at the interface, similarity ratio is the fraction of similar atoms over overlapping atoms. Spearman correlation coefficients: (a): 0.5, (b): 0.4, (c): -0.3, (d):-0.02.

Comparison between our results and Humphris and Kortemme [1]

There is a significant overlap between the data used in the present study, and the one used by Humphris and Kortemme [1]. They used 20 multi-specific proteins, seen in complex with different partners in 65 PDB structures. The 20 clusters are overlapping with our 433 pairs in the following extend:

- cluster 1 correspond to 6 ABAC pairs,
- cluster 4 correspond to 2 ABAC pairs,
- cluster 5 correspond to 3 ABAC pairs,
- cluster 8 correspond to 3 ABAC pairs,
- cluster 9 correspond to 111 ABAC pairs,
- cluster 10 correspond to 1 ABAC pair,
- cluster 13 correspond to 7 ABAC pairs,
- cluster 17 correspond to 1 ABAC pairs,
- cluster 11, 12, 16 and 18 correspond to 130 ABAC pairs.

Other clusters are not represented in our data set. The clusters cover 264 ABAC pairs. The correspondence is established by mapping the SCOP family of the promiscuous proteins from Humphris and Kortemme to the SCOP family of the A and A' domains of the ABAC pairs. Proteins from clusters 11 (Ran), 12 (Ras), 16 (Rac) and 18 (Cdc42) used by Humphris and Kortemme belong to the same SCOP family (52592, G proteins).

In Figure 22 A and B, we report the similarity P-values of all the ABAC pairs covered by Humphris and Kortemme clusters, obtained using Cα interface representation. The color code is the same as in previous figures: red=category A, orange=category M, blue=category E, cyan=category I, green=category S, and white points correspond to domains with no conserved residues. Figures 22 C and D represent P-value histograms for two clusters corresponding to a substantial number of ABAC pairs:

- cluster 9: elastase from [1], corresponding to ABAC pairs of SCOP family 50514=eukaryotic proteases.
- cluster 11/12/16/18: Ran, Ras, Rac and Cdc42 from [1], corresponding to ABAC pairs of SCOP family 5292=G proteins.

Cluster 9 belongs to group 1 delineated in [1], where all interaction partners probably share key interactions residues, and clusters 11, 12, 16 and 18 belong to group 2, where each binding partner prefers its own subset of wild-type residues within the promiscuous binding site. Our results are in good agreement with the distinction between these two groups: the distribution of similarity P-value on the A/A' domains is clearly dominated by low values for cluster 9 (Figure 22 C), whereas it is less clear for cluster 11/12/16/18 (Figure 22 D). It indicates that A/A' binding sites are clearly similar within the pairs of cluster 9, and more variable within the pairs of cluster 11/12/16/18, in agreement with the group classification. Our results provide additional information on the similarity between B and C binding sites.

References

[1] E. L. Humphris and T. Kortemme. Design of multi-specificity in protein interfaces. *PLoS Comput. Biol.*, 3:e164, Aug 2007.

[2] J. Mintseris and Z. Weng. Optimizing protein representations with information theory. *Genome Inform*, 15:160–169, 2004.

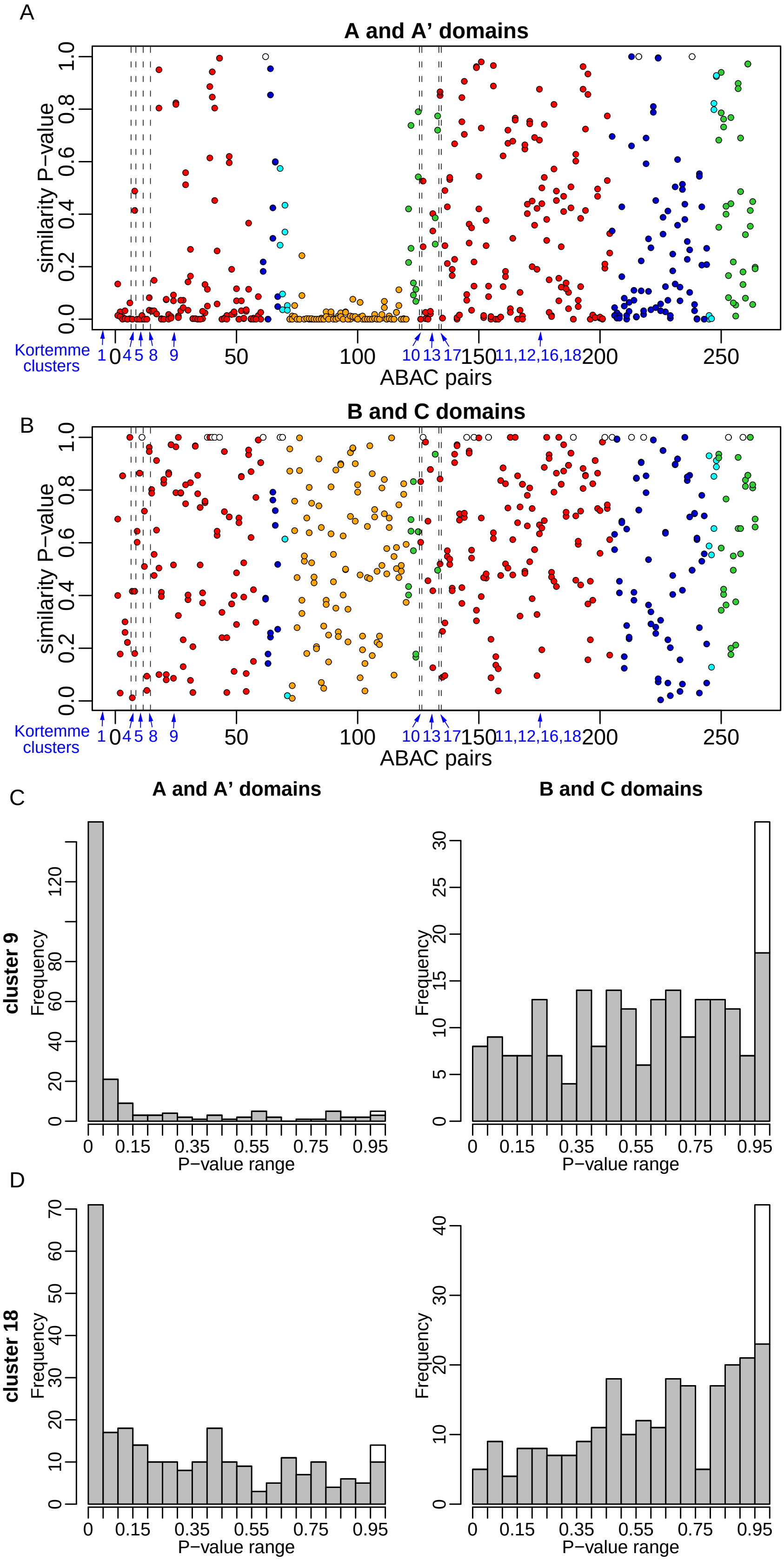

Figure 22: **Comparison between the present study and the work of Humphris and Kortemme.** See text
